# Supplementary material for: Onion-like multicolor thermally activated delayed fluorescent carbon quantum dots for efficient electroluminescent light-emitting diodes
Source: Nat Commun. 2024 Apr 8;15:3043. doi: 10.1038/s41467-024-47372-8 (PMC11001924; doi:10.1038/s41467-024-47372-8)
Supplement: Supplementary file 1 — Supplementary Information [file 41467_2024_47372_MOESM1_ESM.pdf]

Supplementary Information

Onion-like Multicolor Thermally Activated Delayed Fluorescent Carbon Quantum Dots for Efficient Electroluminescent Light-emitting Diodes

Yuxin Shi[1], Yang Zhang[1*], Zhibin Wang[2], Ting Yuan[1], Ting Meng[1], Yunchao Li[1], Xiaohong Li[1], Fanglong Yuan[1*], Zhan'ao Tan[3*] and Louzhen Fan[1*]

[1] Key Laboratory of Theoretical & Computational Photochemistry of Ministry of Education, College of Chemistry, Beijing Normal University, Beijing, 100875, China

[2] College of Physics and Energy, Fujian Normal University, Fuzhou, 350117, China

[3] Beijing Advanced Innovation Centre for Soft Matter Science and Engineering, Beijing University of Chemical Technology, Beijing, 100029, China

E-mail: y.zhang@bnu.edu.cn; flyuan@bnu.edu.cn; tanzhanao@buct.edu.cn; lzfan@bnu.edu.cn

Supplementary Figures 2

Supplementary Tables 26

Supplementary Methods 29

Supplementary References 33

Supplementary Figures

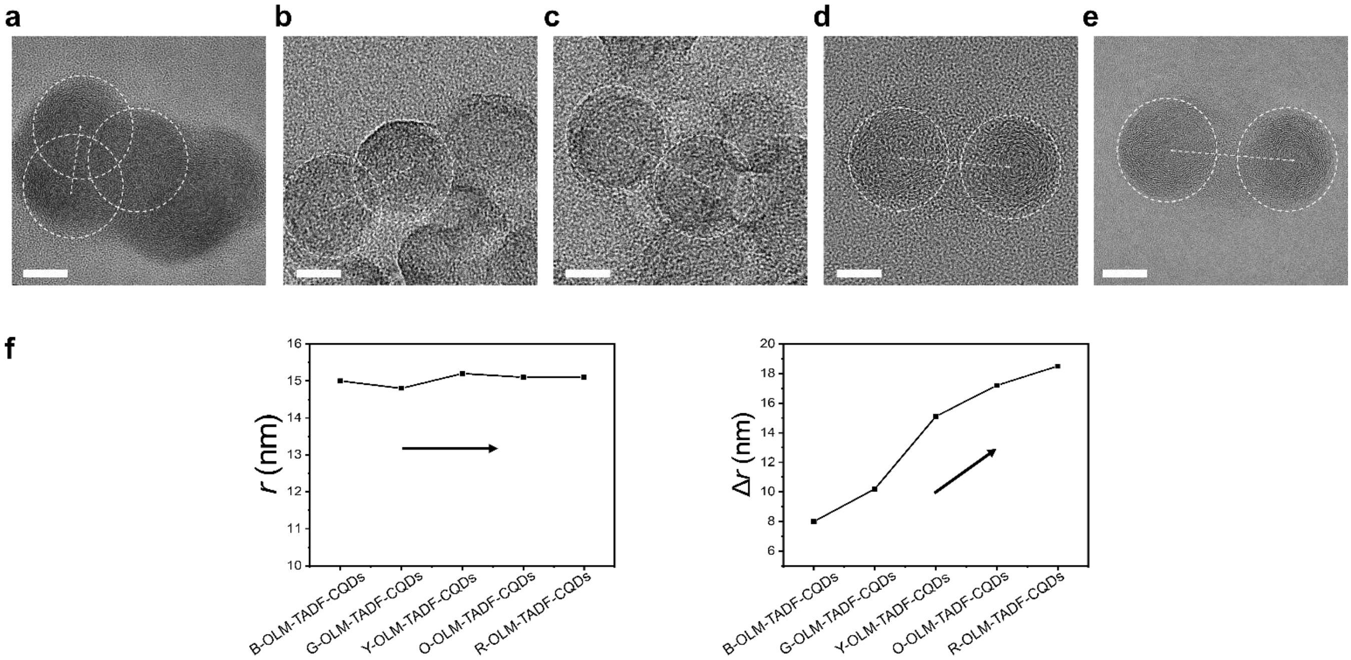

Supplementary Figure 1. The size of OLM-TADF-CQDs with different color. a-e TEM images (Scale bare: 10 nm) and **f** size distributions of blue (B-), green (G-), yellow (Y-), orange (O-) to red (R-) onion-like multicolor thermally activated delayed fluorescence carbon quantum dots (OLM-TADF-CQDs). The variation of the size distribution (*r*) of OLM-TADF-CQDs for different emission is small, but the distance between each OLM-TADF-CQDs gradually increases (Δ*r*).

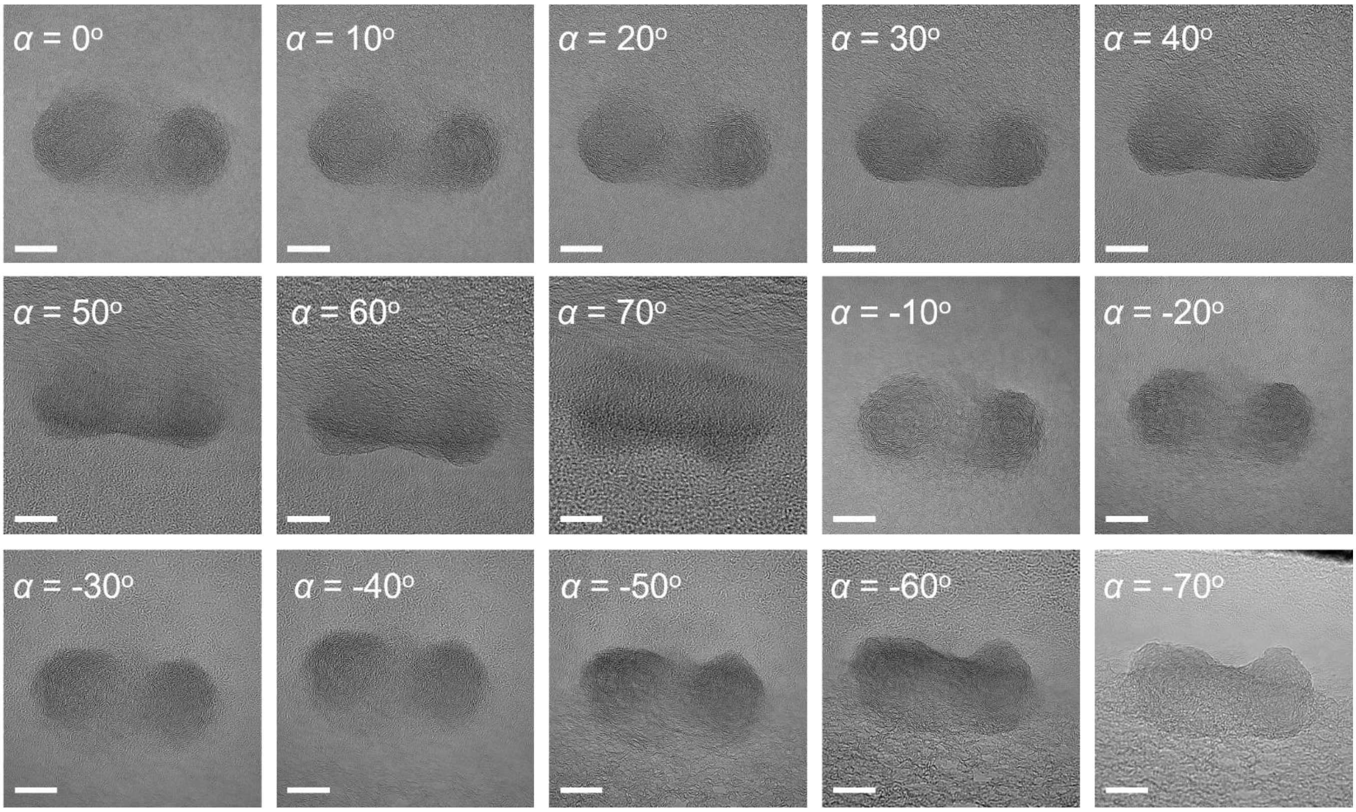

Supplementary Figure 2. STEM images of R-OLM-TADF-CQDs. A tilt series of images of R-OLM-TADF-CQDs recorded in STEM (Scale bare: 10 nm) with different tilt angles (α), including 0°, 10°, 20°, 30°, 40°, 50°, 60°, 70°, –10°, –20°, –30°, –40°, –50°, –60° and –70°.

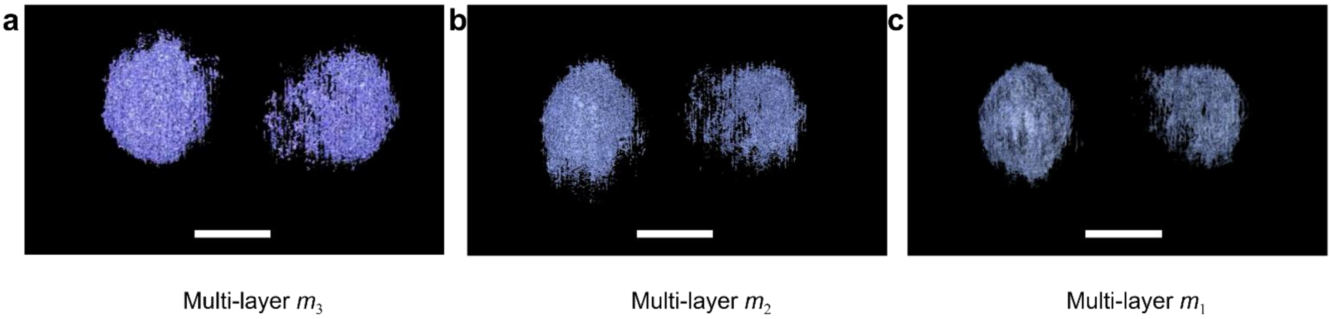

Supplementary Figure 3. 3D model and reconstruction images. The onion-like multi-layered structure is recorded by the STEM tilt series ($\alpha \sim$ −70°–70°) of the layered derivation process, starting from **a** multi-layer m_3 (the third layer), **b** multi-layer m_2 (the second layer), and **c** multi-layer m_1 (the first layer) (Scale bare: 10 nm).

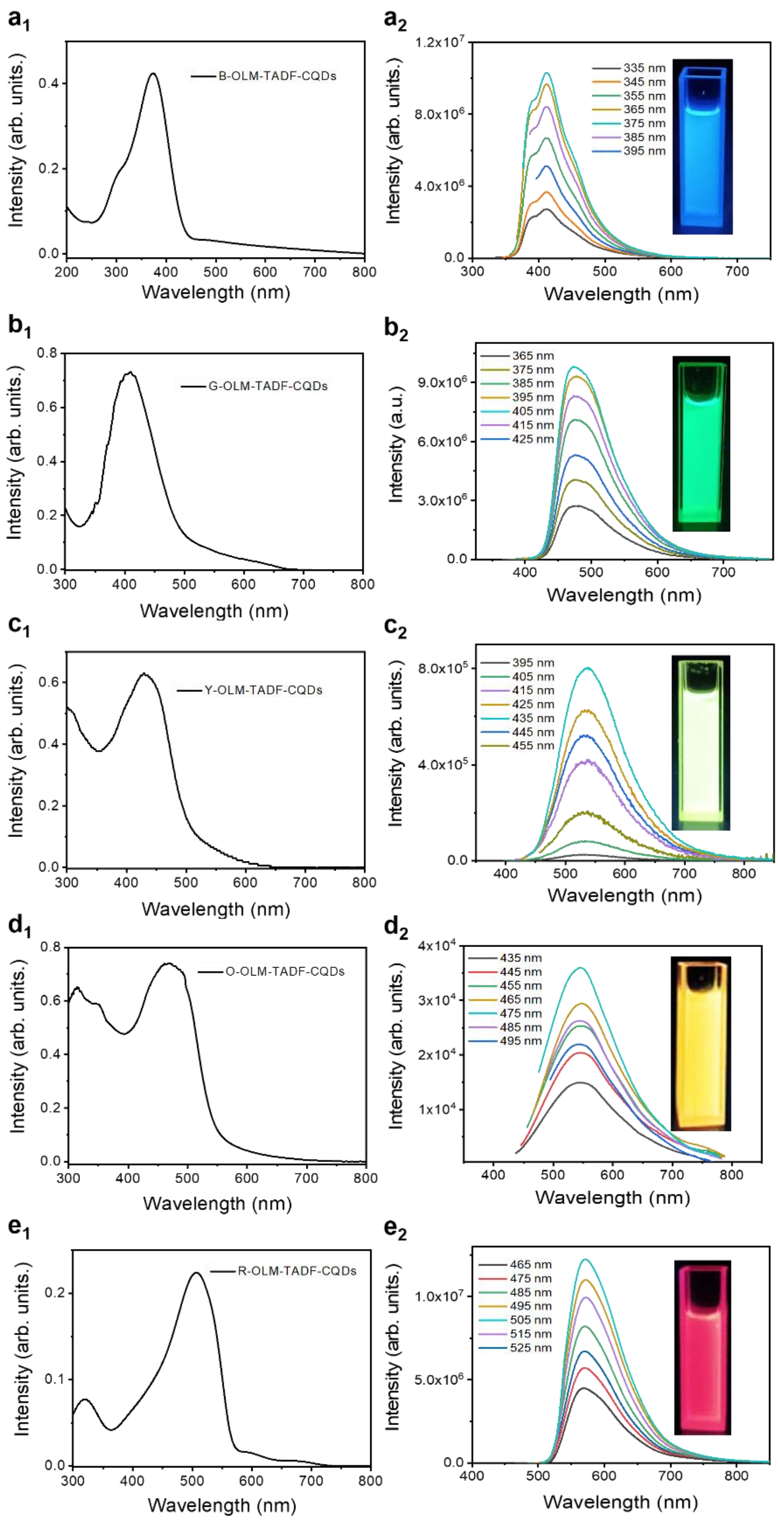

Supplementary Figure 4. Optical properties at solution state. a-e UV-vis absorption spectra and PL spectra of blue (B-), green (G-), yellow (Y-), orange (O-) to red (R-) onion-like multicolor thermally activated delayed fluorescence carbon quantum dots (OLM-TADF-CQDs) at solution state with *o*-dichlorobenzene as the solvent (insets are the optical photographs under UV light (365 nm)).

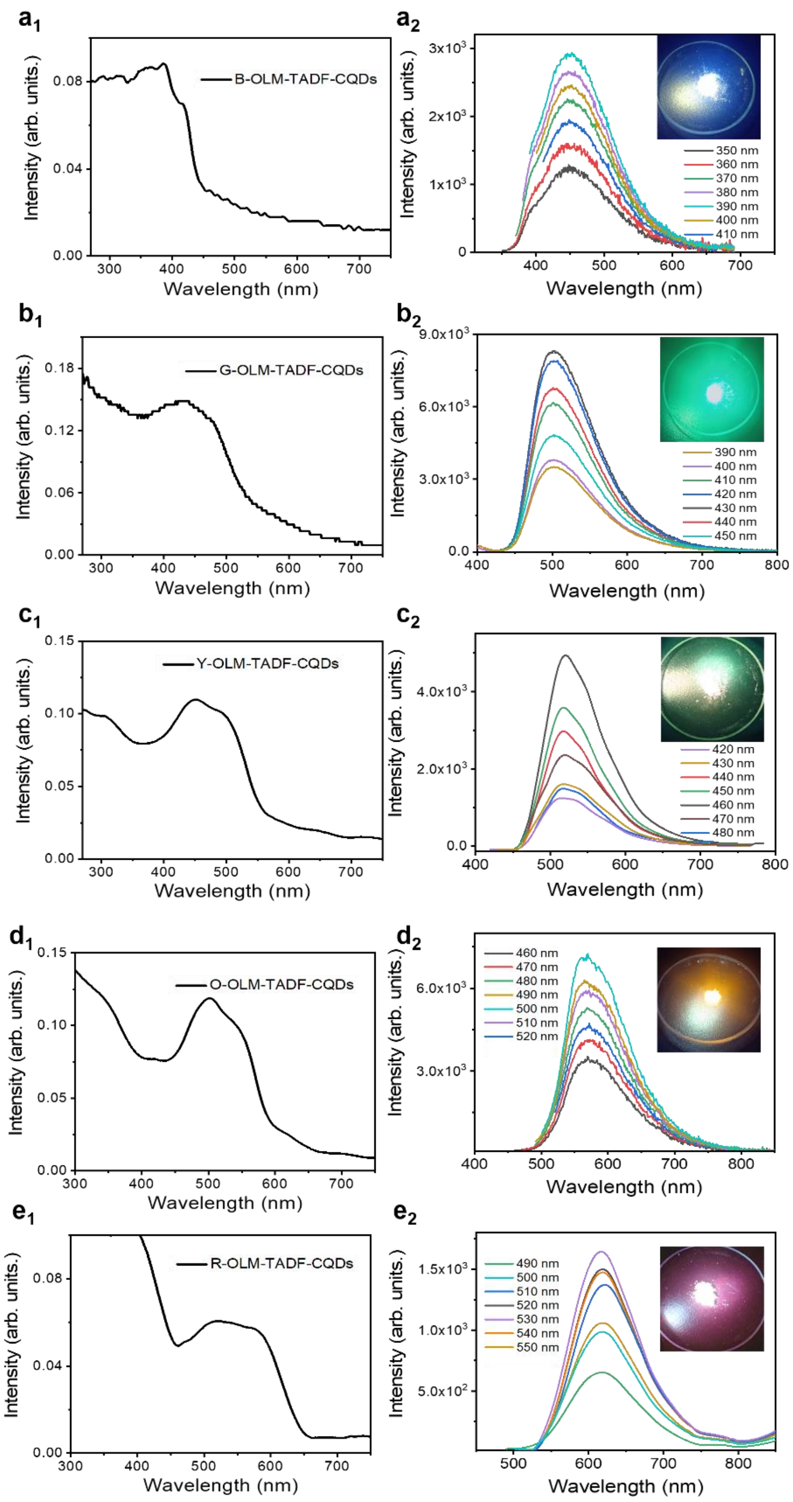

Supplementary Figure 5. Optical properties at solid state. a-e UV-vis absorption spectra and PL spectra of blue (B-), green (G-), yellow (Y-), orange (O-) to red (R-) onion-like multicolor thermally activated delayed fluorescence carbon quantum dots (OLM-TADF-CQDs) at solid state (insets are the optical photographs under UV light (365 nm)).

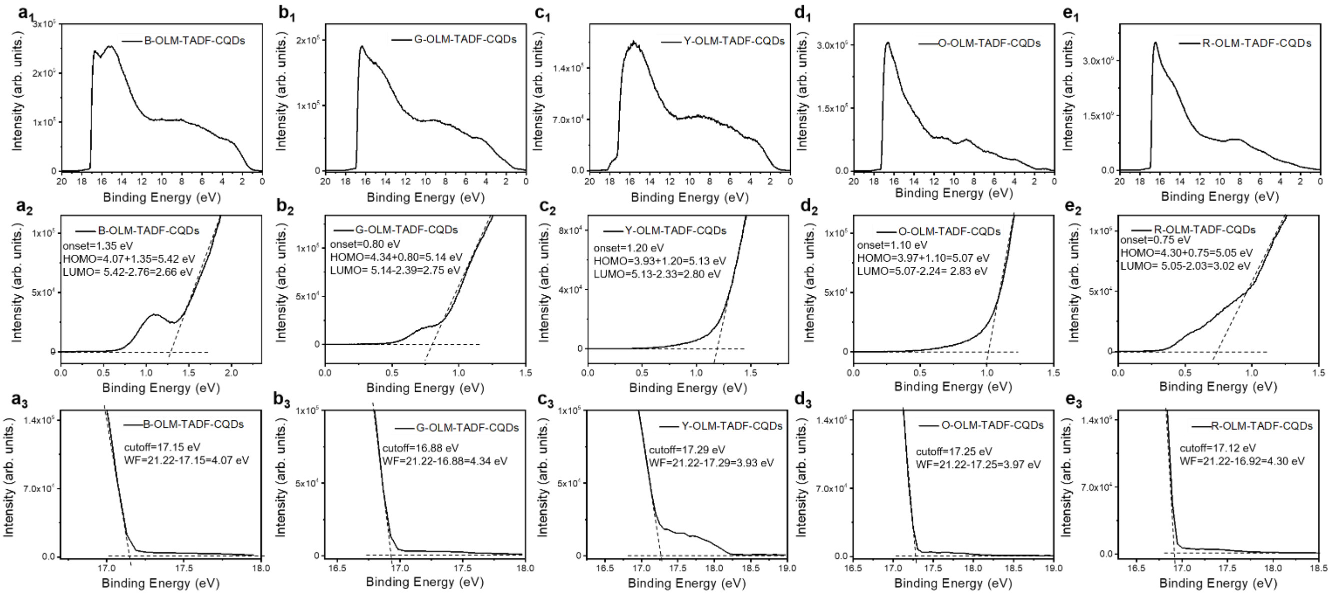

Supplementary Figure 6. UPS for the HOMO and LUMO. a-e UV photoelectron spectroscopy data of blue (B-), green (G-), yellow (Y-), orange (O-) to red (R-) onion-like multicolor thermally activated delayed fluorescence carbon quantum dots (OLM-TADF-CQDs). The work function (WF) is calculated by WF = $h\nu - E_{\text{cutoff}}$ (He I, $h\nu$ = 21.22 eV), the highest occupied molecular orbital (HOMO) is calculated by HOMO = $h\nu - (E_{\text{cutoff}} - E_{\text{onset}})$ and the lowest unoccupied molecular orbital (LUMO) is calculated by HOMO and energy gap.

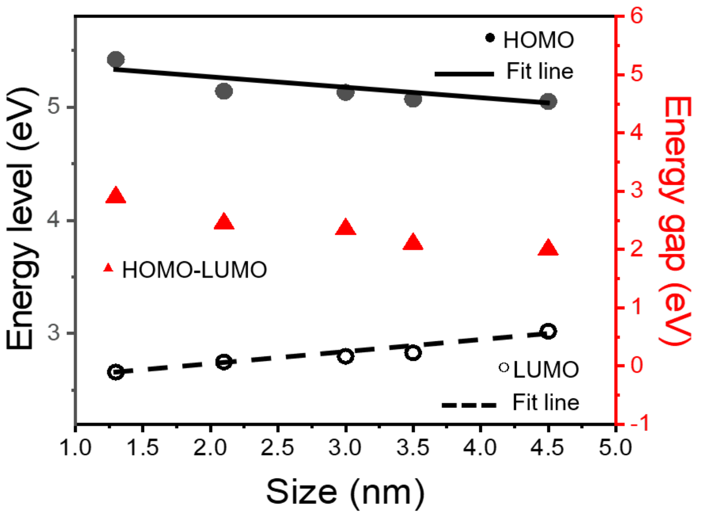

Supplementary Figure 7. The change of HOMO and LUMO energy level. The energy levels of the highest occupied molecular orbital (HOMO) and the lowest unoccupied molecular orbital (LUMO), and energy gap as a function of the size of monomer CQDs are shown. The bandgap energies are calculated using the equation E_g^{opt} = $1240/\lambda_{\text{edge}}$, where λ_{edge} is the onset value of the first excitonic absorption bands in the direction of longer wavelengths.

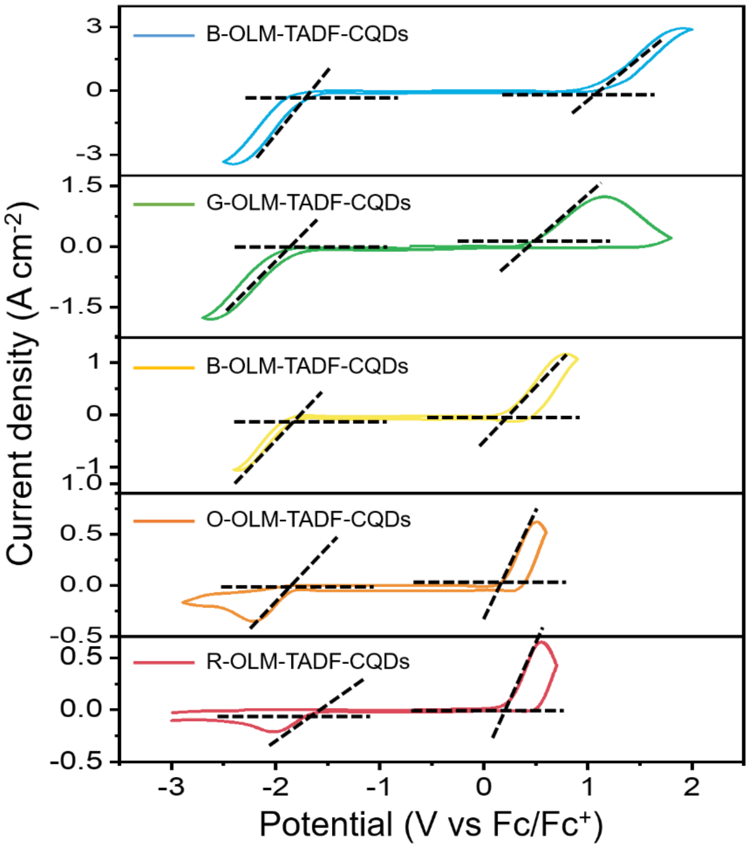

Supplementary Figure 8. Cyclic voltammogram curves for the energy level. Cyclic voltammogram curves of blue (B-), green (G-), yellow (Y-), orange (O-) to red (R-) onion-like multicolor thermally activated delayed fluorescence carbon quantum dots (OLM-TADF-CQDs).

The conventional three-electrode system is used for the cyclic voltammogram measurement of OLM-TADF-CQDs. OLM-TADF-CQDs solution (5 mg mL^{-1}) is dropped onto the surface of a glass carbon electrode, as the working electrode, and the film is allowed to dry at 65 °C for 2 h. Ag/AgCl (saturated KCl) is used as the reference electrode, and a platinum wire is used as the counter electrode. Acetonitrile containing 0.1 M $(Bu)_4NPF_6$ is used as the supporting electrolyte. Before each measurement, N_2 purging is conducted for 15 min to remove dissolved oxygen. The energy levels (E) of HOMO and LUMO are calculated from the onset potentials of oxidation ($E_{\mathrm{ox}}^{\mathrm{onset}}$) and reduction ($E_{\mathrm{red}}^{\mathrm{onset}}$) and by assuming the energy level of ferrocene/ferrocenium (Fc/Fc$^+$) to be −4.8 eV below the vacuum level.

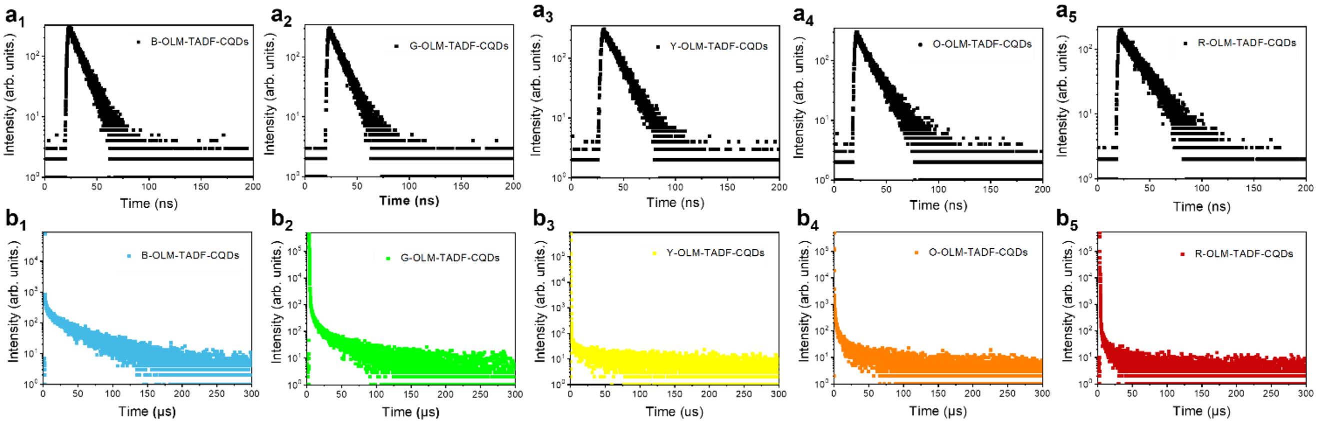

Supplementary Figure 9. Lifetime for OLM-TADF-CQDs. a_1-a_5 Time-resolved PL spectra and **b_1-b_5** time-resolved decay spectra of blue (B-), green (G-), yellow (Y-), orange (O-) to red (R-) onion-like multicolor thermally activated delayed fluorescence carbon quantum dots (OLM-TADF-CQDs) at ambient conditions.

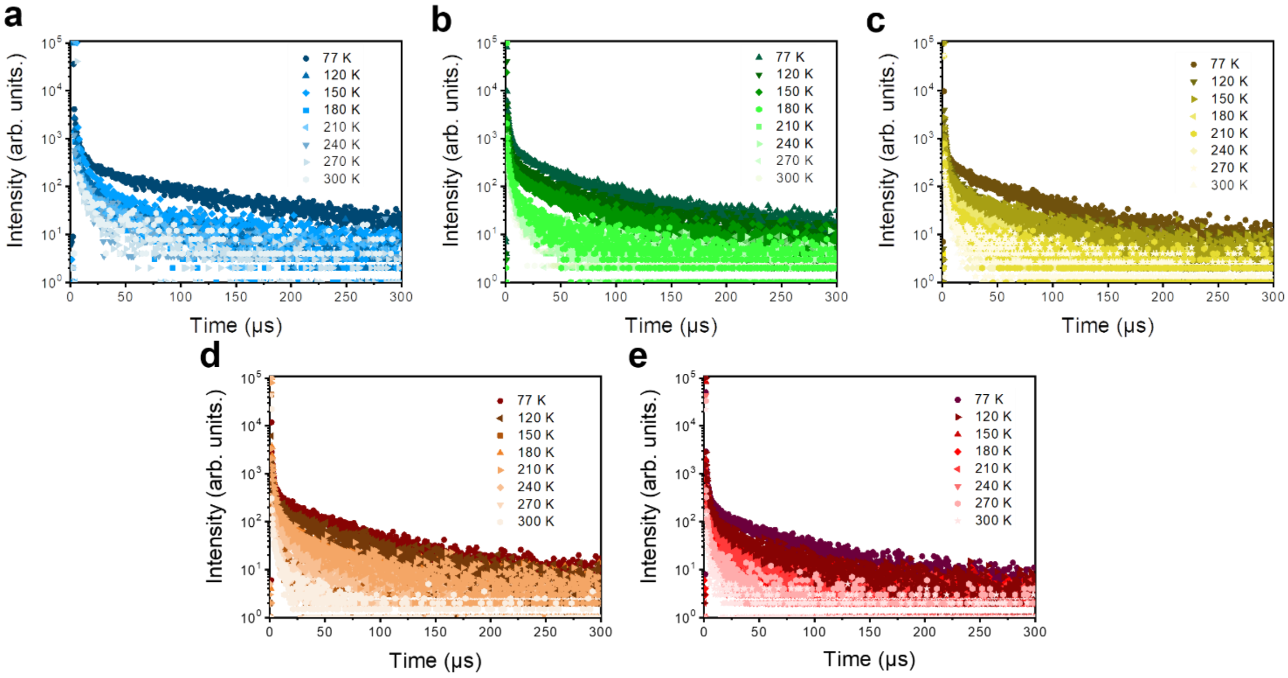

Supplementary Figure 10. Temperature-dependent Lifetime for OLM-TADF-CQDs. a-e Temperature-dependent time-resolved decay spectra (77–300 K) of B-, G-, Y-, O-, and R-OLM-TADF-CQDs.

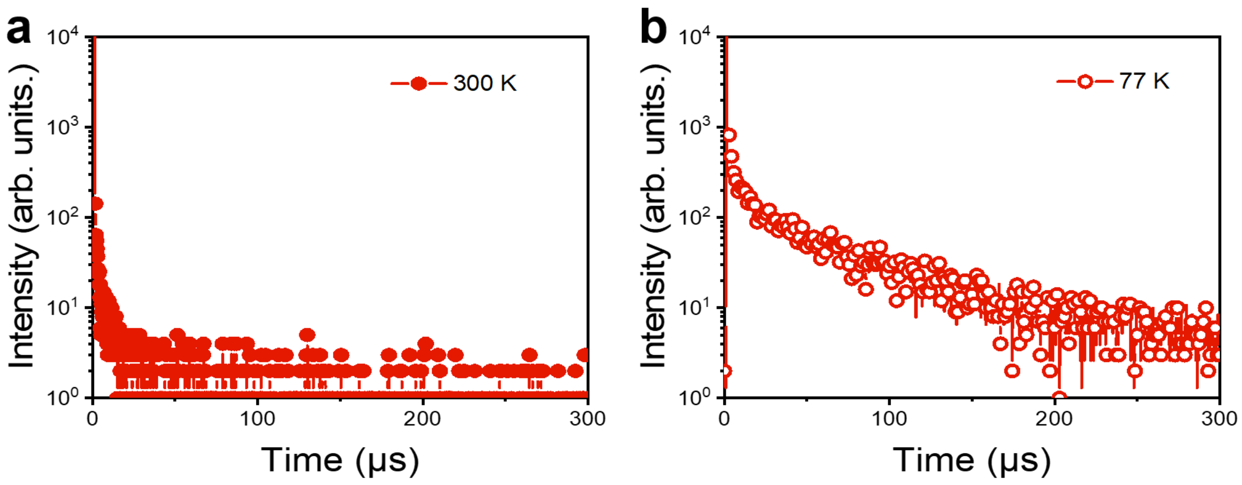

Supplementary Figure 11. The long Lifetime for R-OLM-TADF-CQDs. Temperature-dependent time-resolved decay spectra at **a** 300 K and **b** 77 K of R-OLM-TADF-CQDs.

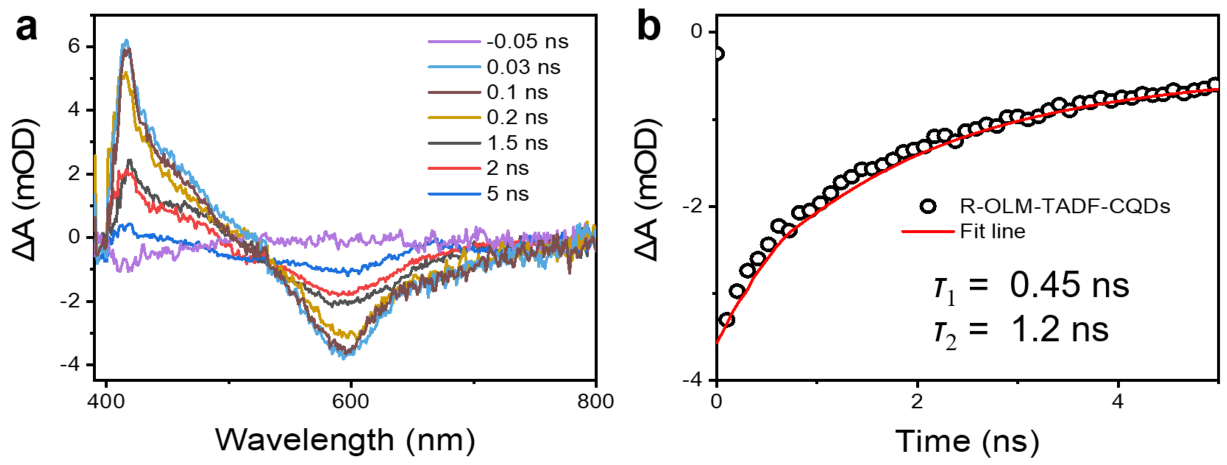

Supplementary Figure 12. Transient absorption spectra and dynamic curves. a Transient absorption spectrum of red onion-like multicolor thermally activated delayed fluorescence carbon quantum dots (R-OLM-TADF-CQDs) with a pump wavelength of 400 nm at indicated delay times (τ). The positive signals at 400–500 nm can be ascribed to the excited-state absorption, and the negative signals at 500–700 nm correspond to the ground state bleaching and stimulate emission. **b** Bleach signal kinetic for pump wavelength of 400 nm and probe wavelength of 580 nm.

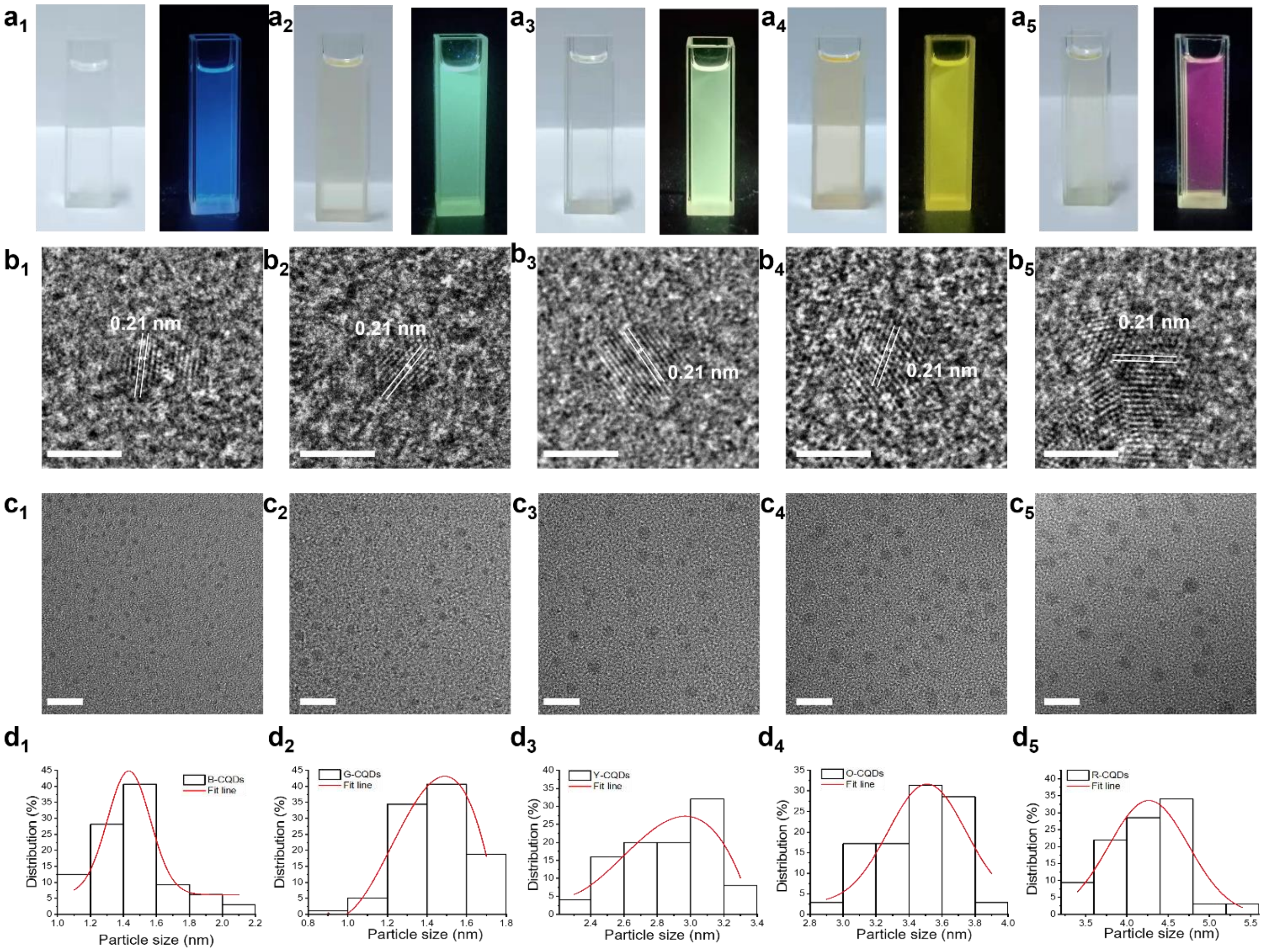

Supplementary Figure 13. Optical properties and size of monomer CQDs. a_1-a_5 Optical photographs under sunlight and UV light (365 nm), **b_1-b_5** TEM images (Scale bare: 2 nm), **c_1-c_5** the corresponding high-resolution TEM images (Scale bare: 10 nm), and **d_1-d_5** size distribution maps of blue (B-), green (G-), yellow(Y-), orange (O-), and red (R-) carbon quantum dots (CQDs). The CQDs are prepared by refluxing the as-prepared blue, green, yellow, orange, and red OLM-TADF-CQDs with sulfuric acid (10 mL) in a round bottom flask at 110 °C for 3 h.

As shown in the TEM images, monomer CQDs with blue, green, yellow, orange, and red fluorescence exhibited average sizes of approximately 1.3 nm, 2.1 nm, 3.0 nm, 3.5 nm, and 4.5 nm, respectively. Their typical high-resolution TEM images further indicate the high crystallinity of monomer CQDs.

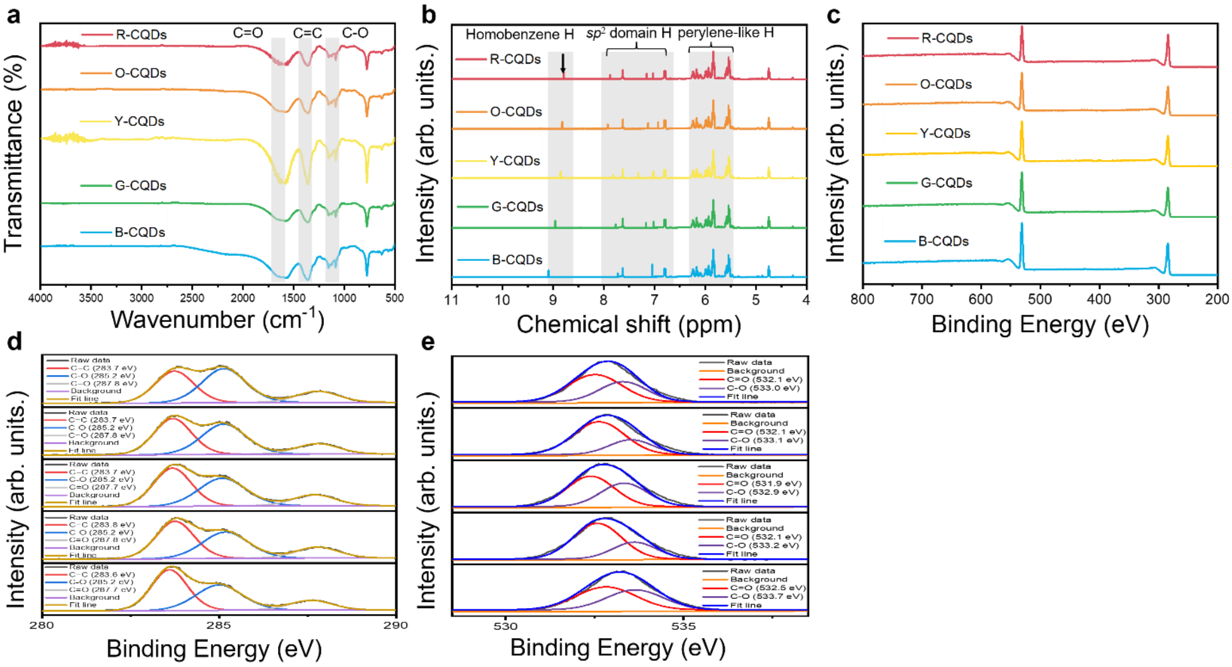

Supplementary Figure 14. Structure characterizations of monomer CQDs. a FT-IR, **b** ^{1}H NMR, and **c** XPS spectra, including **d** high-resolution C1s and **e** O1s XPS spectra of blue (B-), green (G-), yellow(Y-), orange (O-), and red (R-) carbon quantum dots (CQDs).

FT-IR characterizations show that the characteristic stretching vibration bands of C=O, C=C, and C–O are observed at 1630 cm^{-1}, 1350 cm^{-1}, and 1240 cm^{-1}, respectively. XPS spectra demonstrate that the monomer CQDs are primarily comprised of C and O. The deconvoluted high-resolution XPS spectra of C 1s confirm the presence of C=O, C–O, and C=C. Moreover, in the ^{1}H-NMR spectra (methanol-d_4, ppm), the signals in the ranges of 5–7 ppm, 7–8 ppm and 9–10 ppm correspond to the H of perylene-like molecules, the carbon core, and 1,3,5-benzenetricarboxylate-like molecules, respectively.

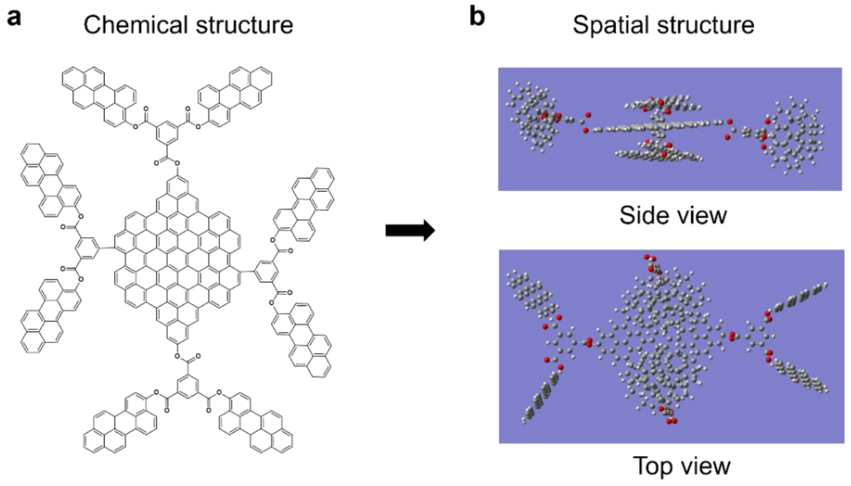

Supplementary Figure 15. Structure of monomer CQDs. a Chemical and **b** spatial structure of monomer CQDs. The theoretical simulation is conducted using the MM2 method.

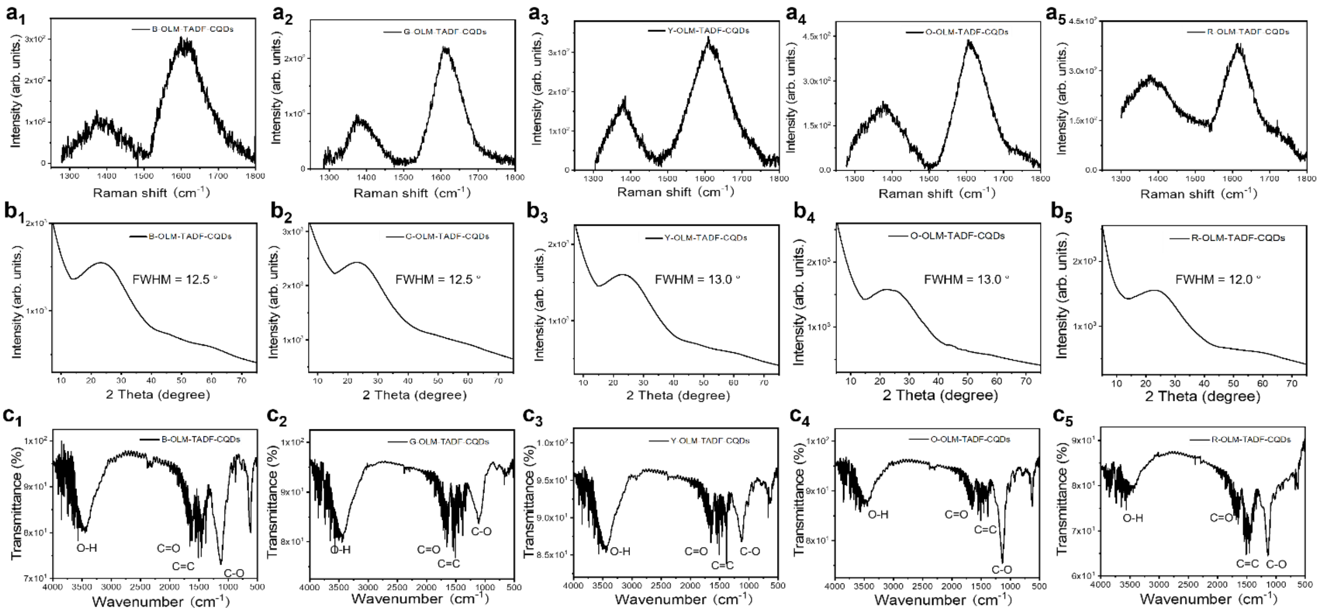

Supplementary Figure 16. Structure characterizations of OLM-TADF-CQDs. a Raman, **b** XRD, and **c** FT-IR spectra of blue (B-), green (G-), yellow (Y-), orange (O-) to red (R-) onion-like multicolor thermally activated delayed fluorescence carbon quantum dots (OLM-TADF-CQDs).

In comparison with previous reports, the full width at half maximum (FWHM) for XRD peaks of OLM-TADF-CQDs (~12°) are larger than those of reported carbon dots with high crystallinity (~8°–10°), which can be ascribed to the self-assembly process. These values are smaller than those of reported carbon dots after aggregation or assembly (~14°), which can be attributed to the more ordered stacking of the assembled 3D onion-like structure.

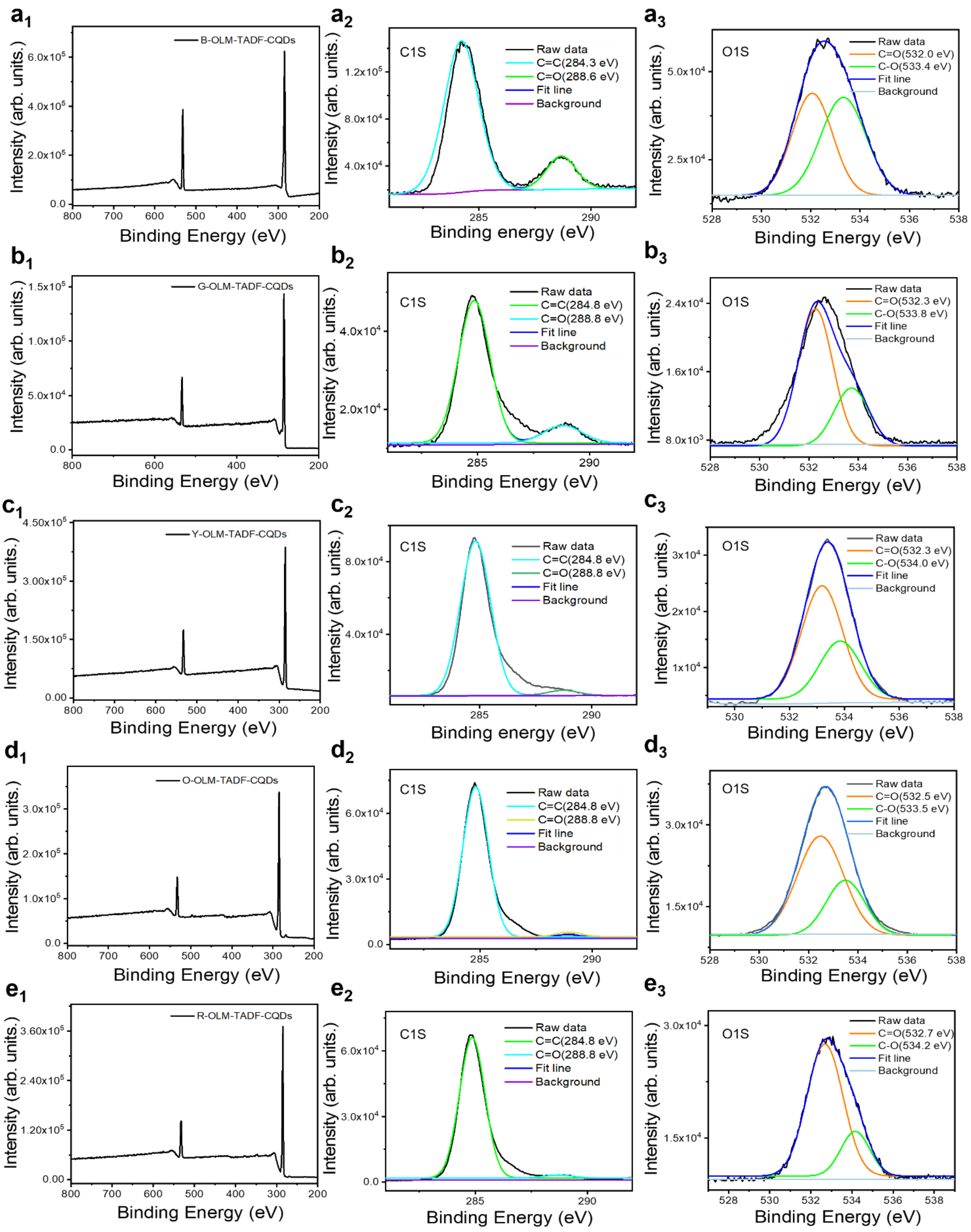

Supplementary Figure 17. XPS spectra. a-e XPS spectra, high-resolution C1s and O1s XPS spectra of blue (B-), green (G-), yellow (Y-), orange (O-) to red (R-) onion-like multicolor thermally activated delayed fluorescence carbon quantum dots (OLM-TADF-CQDs).

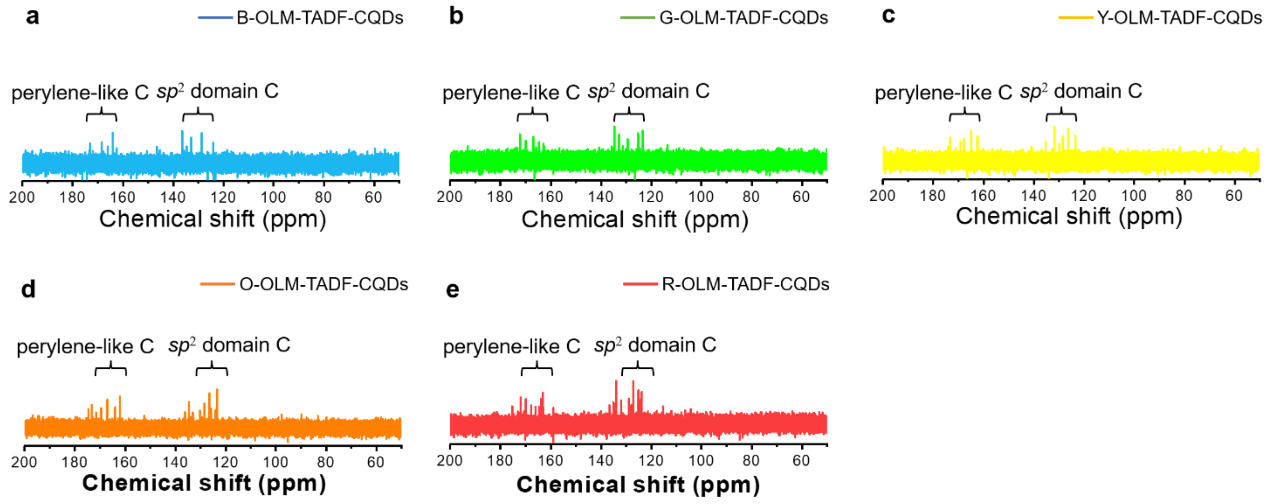

Supplementary Figure 18. NMR spectra. a-e ^{13}C-NMR spectra of blue (B-), green (G-), yellow (Y-), orange (O-) to red (R-) onion-like multicolor thermally activated delayed fluorescence carbon quantum dots (OLM-TADF-CQDs).

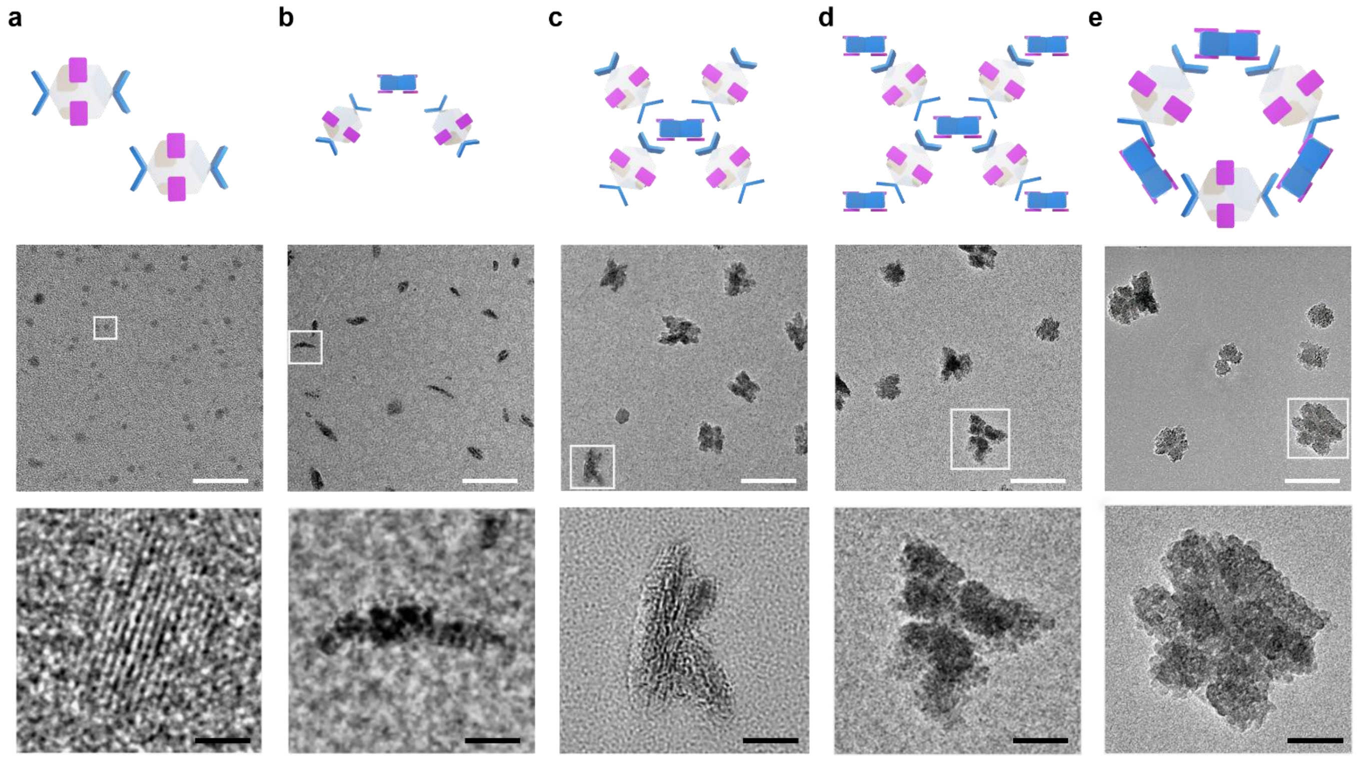

Supplementary Figure 19. The assembly process of OLM-TADF-CQDs. a-e Structure models, large-scale TEM images (Scale bare: 50 nm), and the corresponding high-resolution TEM images (Scale bare: 10 nm) of R-OLM-TADF-CQDs at different assembled structures.

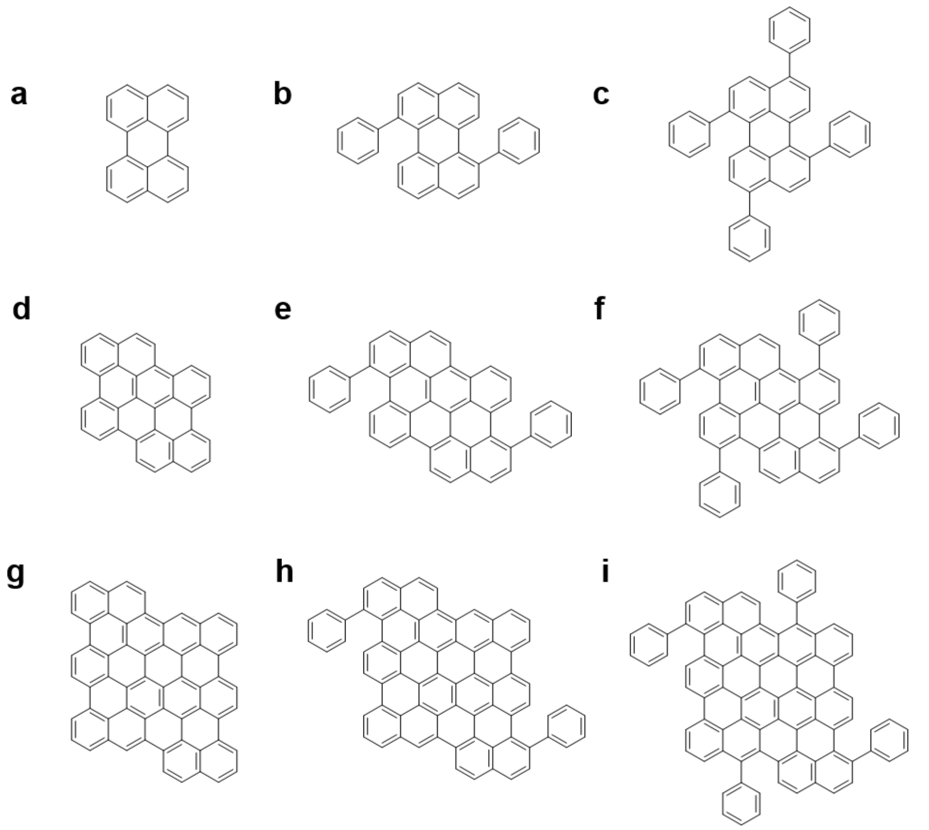

Supplementary Figure 20. The calculated models of monomer CQDs. The model CQDs consisting of five, ten, and twenty benzene rings 1) without any groups (named **a** CQDs **1**, **d** CQDs **4**, **g** CQDs **7**, respectively), 2) with two benzene molecules (named **b** CQDs **2**, **e** CQDs **5**, **h** CQDs **8**, respectively), and 3) with four benzene molecules around the CQDs (named **c** CQDs **3**, **f** CQDs **6**, **i** CQDs **9**, respectively).

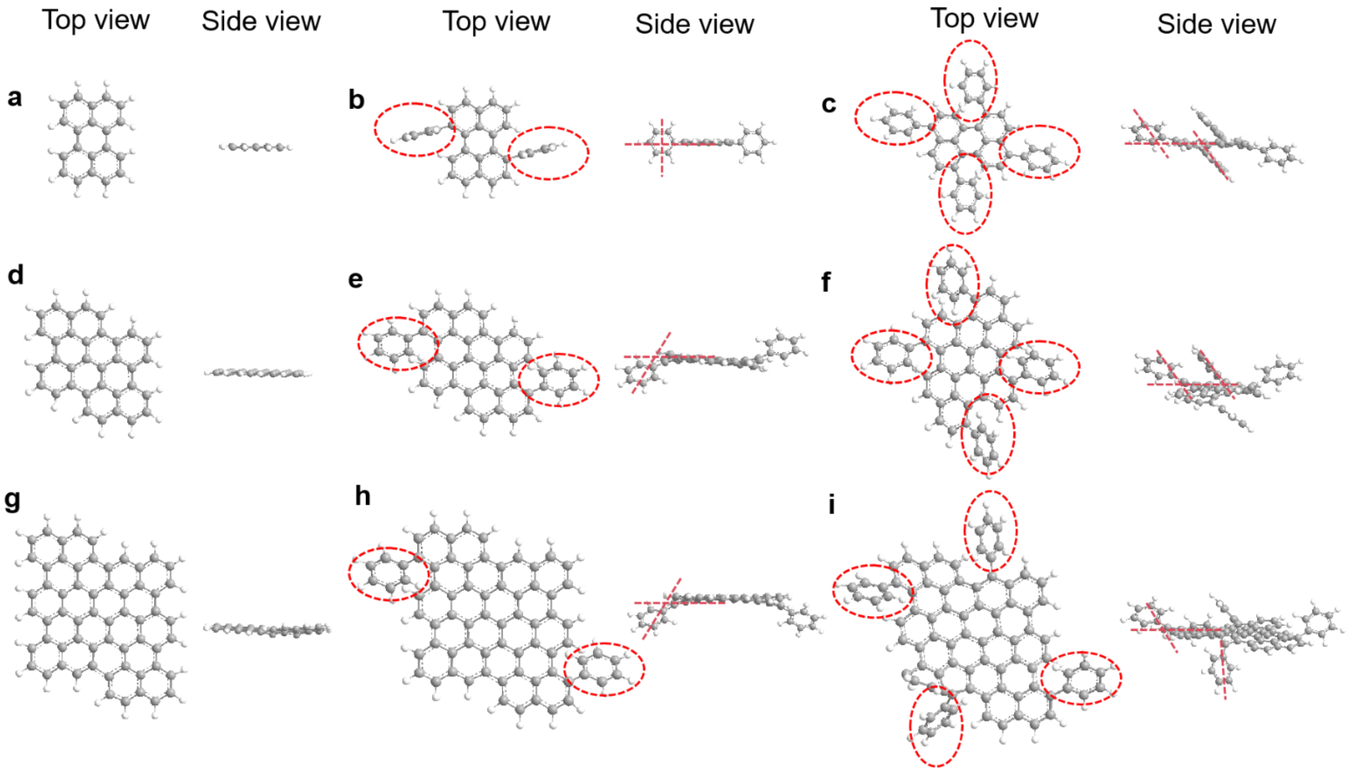

Supplementary Figure 21. The ball-and-stick models of monomer CQDs. Ball-and-stick models (top view and side view) of CQDs consisting of five, ten and twenty benzene rings 1) without any groups (named **a** CQDs **1**, **d** CQDs **4**, **g** CQDs **7**, respectively), 2) with two benzene molecules (named **b** CQDs **2**, **e** CQDs **5**, **h** CQDs **8**, respectively), and 3) with four benzene molecules around the CQDs (named **c** CQDs **3**, **f** CQDs **6**, **i** CQDs **9**, respectively).

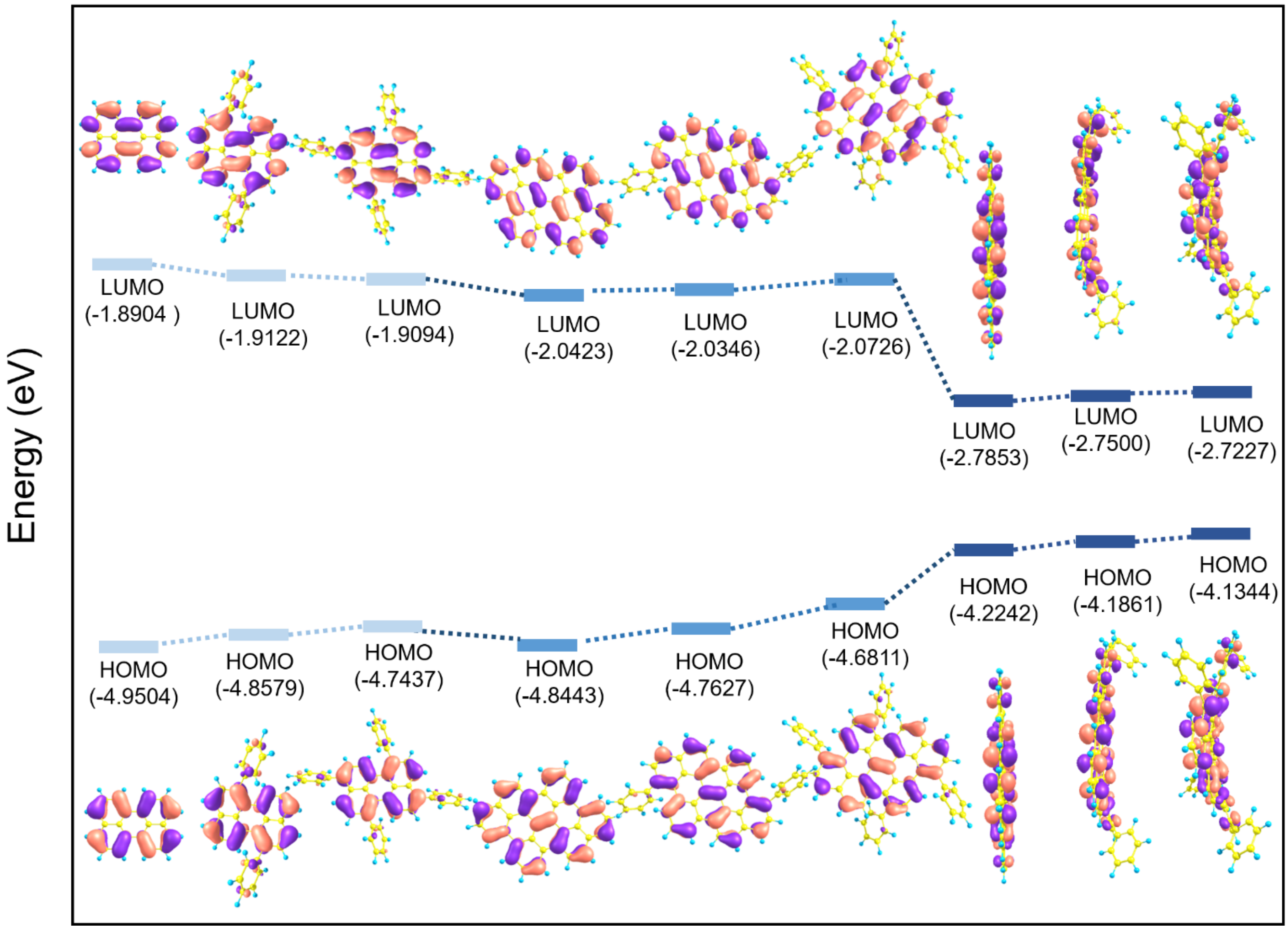

Supplementary Figure 22. Energy level and orbital overlap calculation results. Structural models of monomer CQDs: pure CQDs comprising five, ten, and twenty benzene rings 1) without any groups, 2) with two benzene molecules, and 3) with four benzene molecules around the CQDs. Schematic of the calculated energy levels of the highest occupied molecular orbital (HOMO) and the lowest unoccupied molecular orbital (LUMO).

For CQDs **1**, CQDs **4**, and CQDs **7**, the singlet-triplet energy gap (ΔE (S_1/T_1)) considerably increases with the gradual increase in the sizes of CQDs. However, considering the small singlet-triplet SOC (ξ (S_1, T_1)), which is as low as approximately 0.1 cm^{-1}, this emission is observed only in the singlet state. The modification of two benzene molecules around the CQDs, namely, CQDs **2**, CQDs **5**, and CQDs **8**, affect a decrease in ΔE (S_1/T_1) of approximately 0.50 cm^{-1}. The ξ (S_1, T_1) exhibit a significant increase. Furthermore, adjusting the attachment site and number of benzene molecules to CQDs change the ΔE (S_1/T_1) and increase ξ (S_1, T_1) of CQDs **3**, CQDs **6**, and CQDs **9**. From CQDs **1** to CQDs **9**, ξ (S_1, T_1) significantly increases with the change of functional groups around carbon core, indicating that benzene molecules around the CQDs substantially contribute to triplet emission.

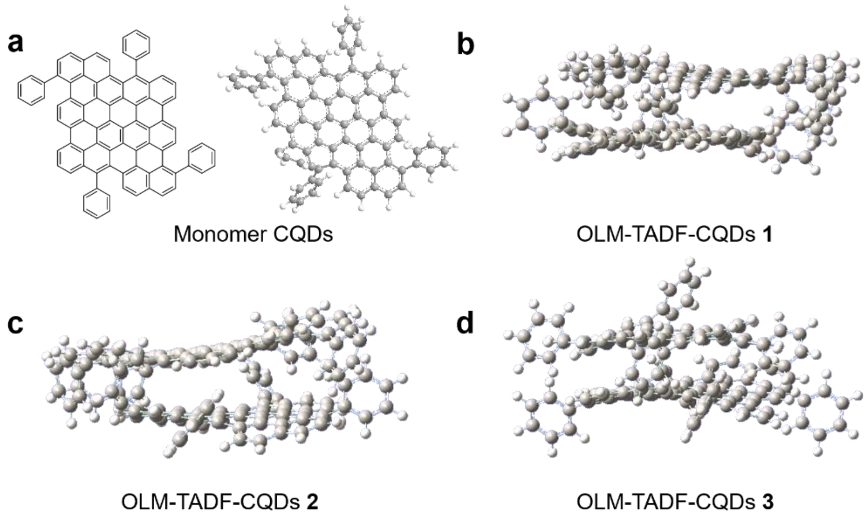

Supplementary Figure 23. The assembled models between monomer CQDs. a Model monomer carbon quantum dots (CQDs) and ball-and-stick models of assembled model structures through two monomer CQDs **b** with π_B–π_B and π_{CQDs}–π_{CQDs} conjugate interactions (named onion-like multicolor thermally activated delayed fluorescence carbon quantum dots **1** (OLM-TADF-CQDs **1**)), **c** only π_B–π_B (named OLM-TADF-CQDs **2**), **d** and only π_{CQDs}–π_{CQDs} (named OLM-TADF-CQDs **3**).

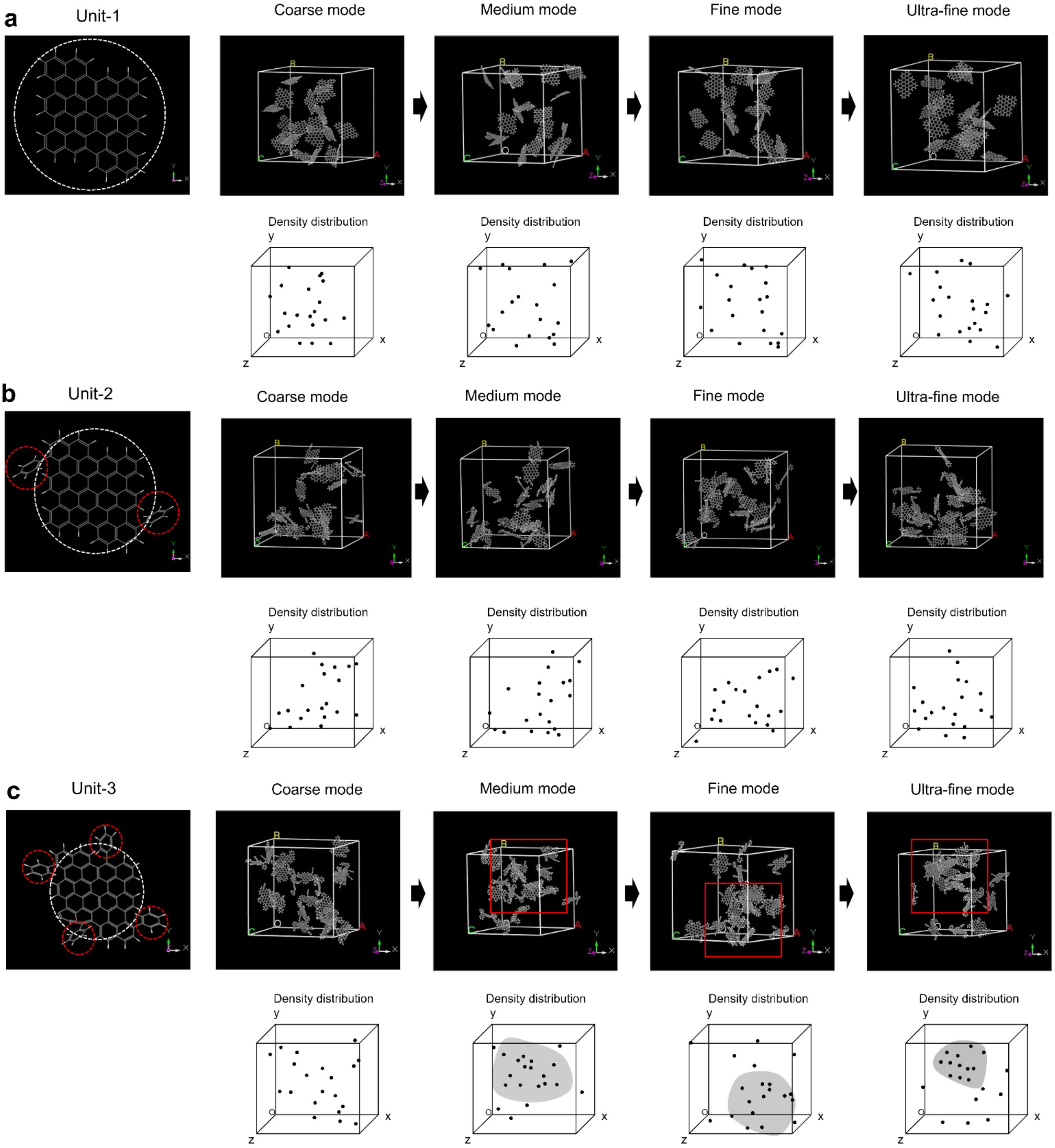

Supplementary Figure 24. Molecular dynamics simulation. There are two structural units: **a** CQDs **7**, **b** CQDs **8**, and **c** CQDs **9** (the carbon core is highlighted as white dotted line, and the functional groups are highlighted as red dotted line). Monomer CQDs are constructed in a cubic water box of 50 × 50 × 50 Å^3 and equilibrated by 200 ns of MD simulation. Twenty monomer units each are packed into a confined layer. The constant pressure and temperature ensemble simulations are conducted at 1 atm and 298 K, maintained with the Berendsen barostat and Andersen thermostat, respectively, producing the target density of 0.1 g cm^{-3}.

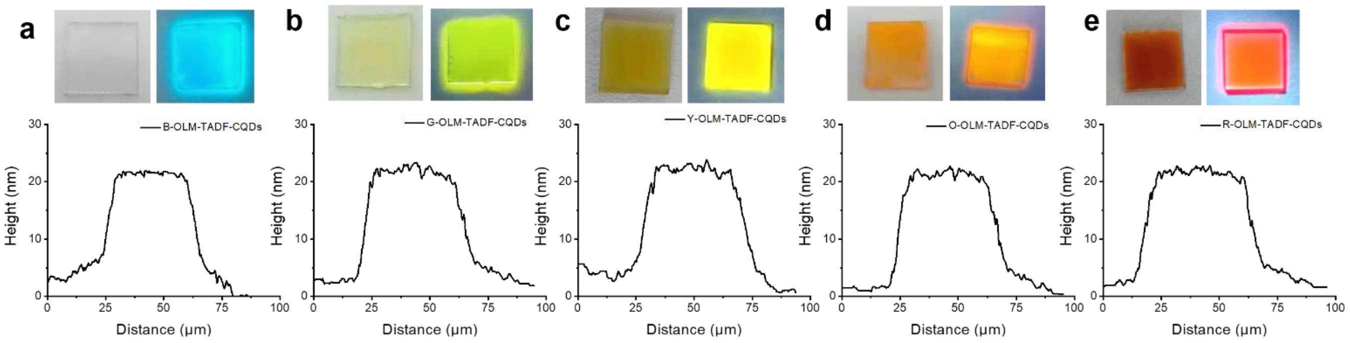

Supplementary Figure 25. The films of OLM-TADF-CQDs. a-e Optical photographs under sunlight and UV light (365 nm) and height curves of the films based on blue (B-), green (G-), yellow (Y-), orange (O-) to red (R-) onion-like multicolor thermally activated delayed fluorescence carbon quantum dots (OLM-TADF-CQDs).

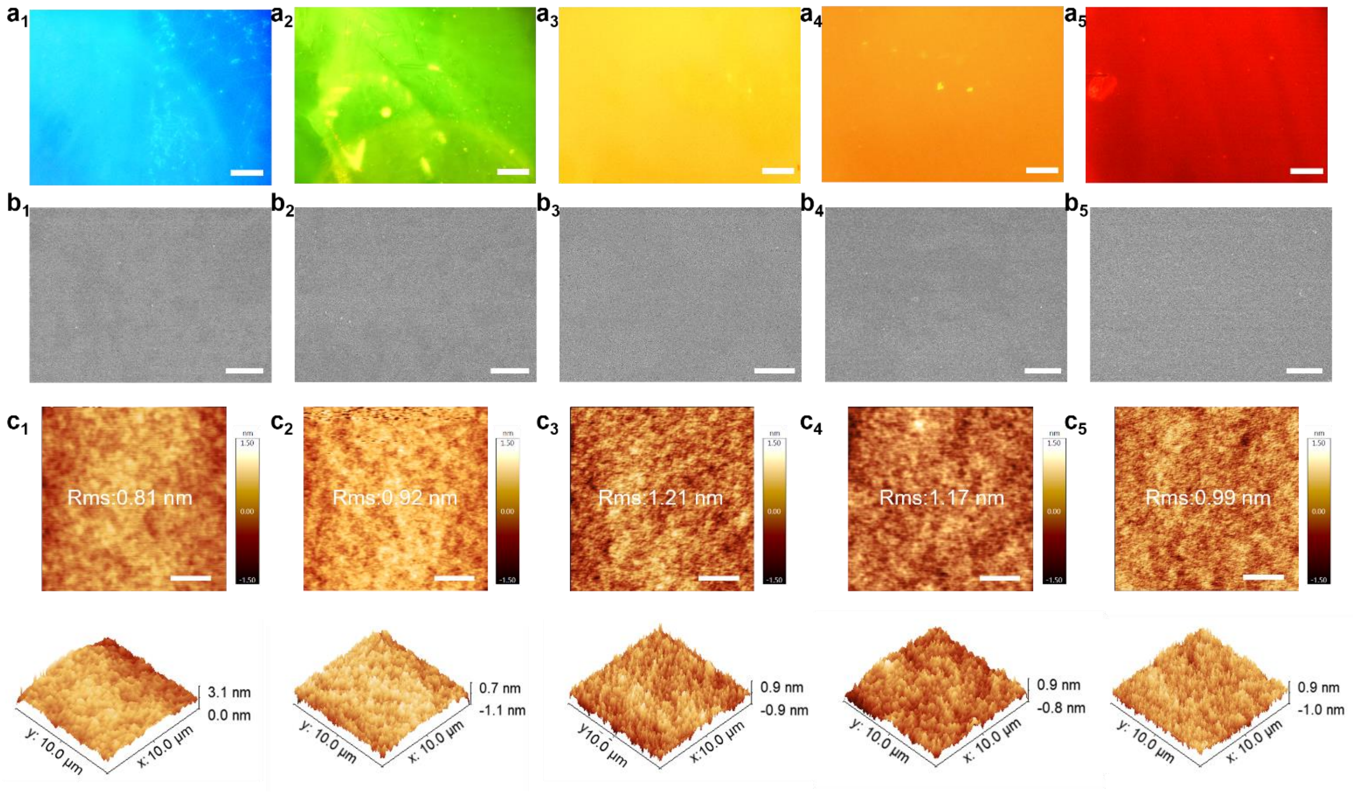

Supplementary Figure 26. The film morphology of OLM-TADF-CQDs. a_1-a_5 Fluorescence microscope images (Scale bare: 20 μm), **b_1-b_5** SEM images (Scale bare: 2 μm), and **c_1-c_5** AFM images and surface roughness (Root Mean Square: RMS) (Scale bare: 2 μm) of B-, G-, Y-, O-, and R-OLM-TADF-CQDs.

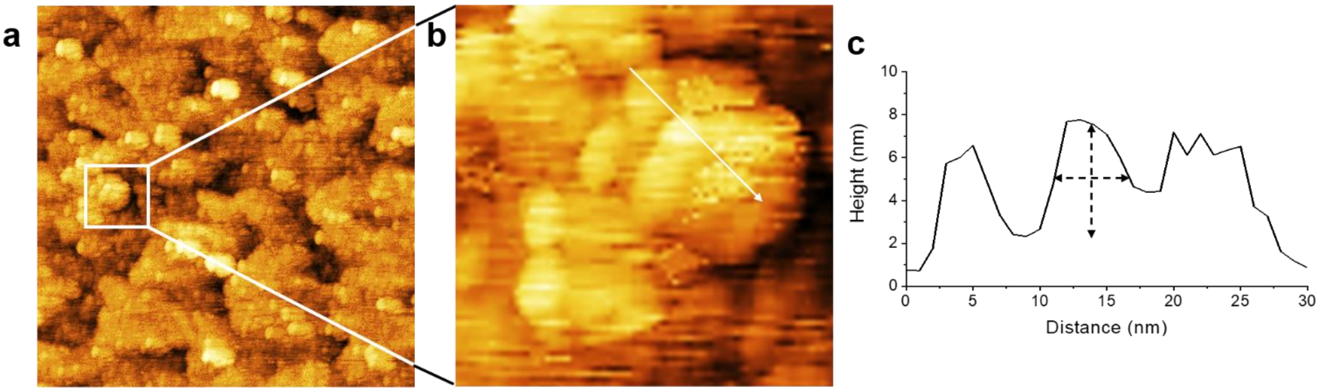

Supplementary Figure 27. The high-resolution morphology of OLM-TADF-CQDs. a High-resolution AFM image of a wide area (300 nm × 300 nm), **b** a selected area from the scan (50 nm × 50 nm), and **c** height curve of R-OLM-TADF-CQD film (at color bar: 0.8–7.5 nm).

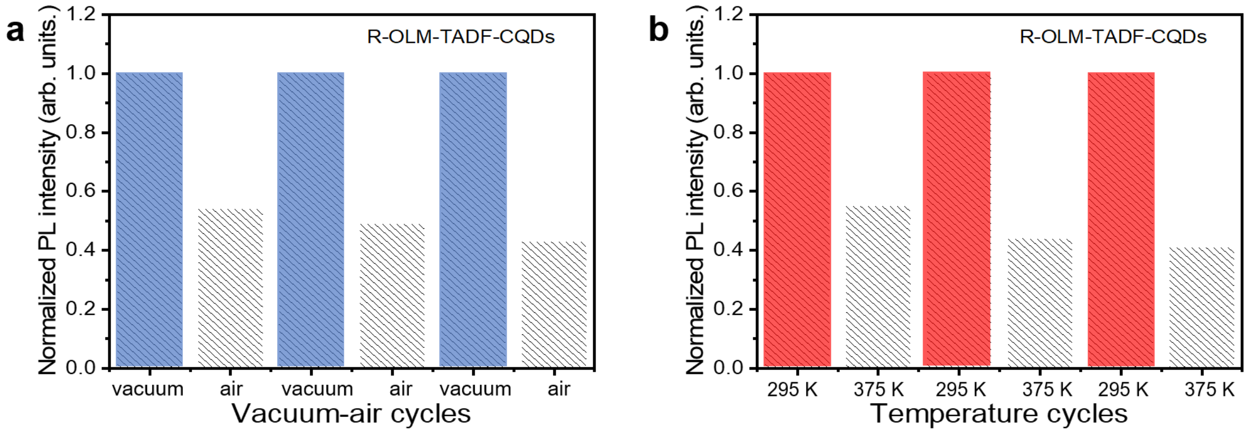

Supplementary Figure 28. The oxygen and temperature stability of films. a Reversible PL quenching by exposure to oxygen of red onion-like multicolor thermally activated delayed fluorescence carbon quantum dot (R-OLM-TADF-CQD) films. Change in PL intensity of a thin film under sequential vacuum-air cycles, normalized to the intensity of the first measurement (vacuum of 10^{-5} mBar in the glove box). Samples stabilize for 10 minutes under vacuum or in air prior to each measurement. The reversibility implicates triplets in the PL pathway, since these can quench by transfer to the triplet ground state of molecular oxygen. **b** Reversible PL quenching of R-OLM-TADF-CQDs films for temperature. Change in PL intensity of a thin film under sequential temperature cycles, normalized to the intensity of the first measurement. Samples stabilize for 10 minutes under 295 K or 375 K prior to each measurement.

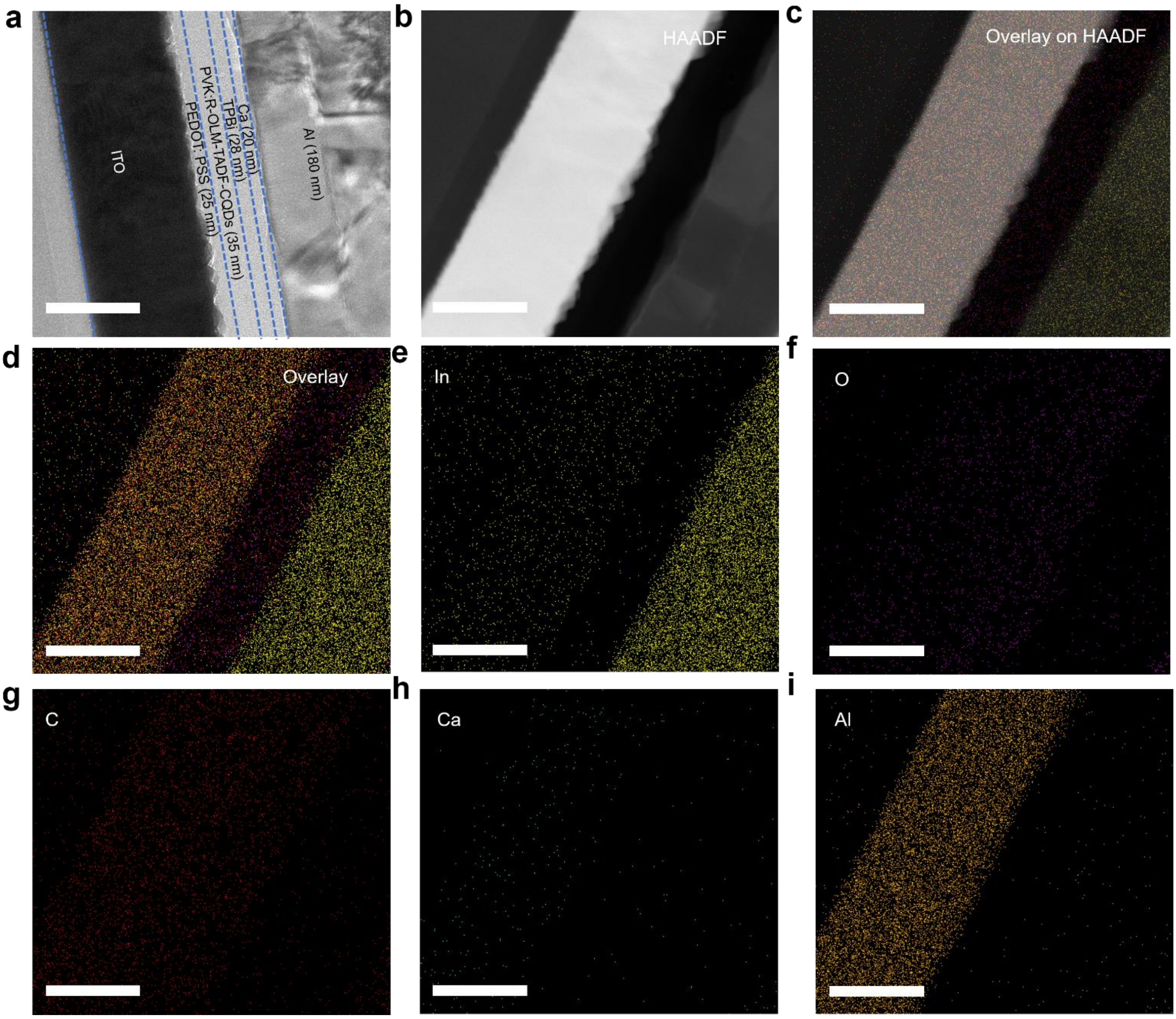

Supplementary Figure 29. The cross-sectional TEM images of devices. a-c The cross-sectional TEM images and **d-i** corresponding energy dispersive X-ray (EDX) mapping images of LEDs based on R-OLM-TADF-CQDs (Scale bars: 150 nm) (ITO: Indium tin oxide, PEDOT:PSS: poly(3,4-ethylenedioxythiophene):poly(styrene-sulfonate), PVK: poly(N-vinyl carbazole), OLM-TADF-CQDs: onion-like multicolor thermally activated delayed fluorescence carbon quantum dots, TPBi: 1,3,5-tris(N-phenylbenzimidazol-2-yl) benzene).

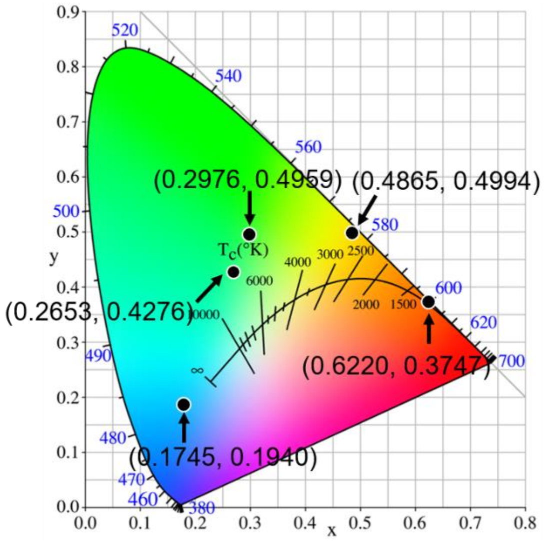

Supplementary Figure 30. The CIE coordinates. CIE coordinates of LEDs based on B-, G-, Y-, O-, and R-OLM-TADF-CQDs are at (0.1745, 0.1940), (0.2653, 0.4276), (0.2976, 0.4959), (0.4865, 0.4994), and (0.6220, 0.3747), respectively.

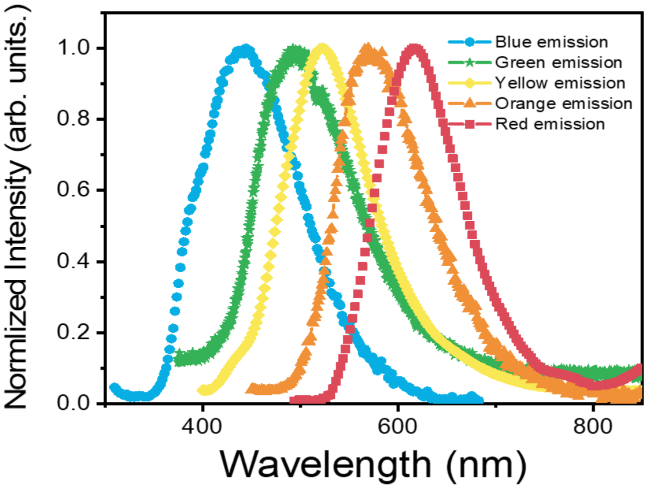

Supplementary Figure 31. PL spectra of OLM-TADF-CQDs as the active layer. The spectra of blue (excitation wavelength, λ_{Ex}: 300 nm), green (λ_{Ex}: 360 nm), yellow (λ_{Ex}: 390 nm), orange (λ_{Ex}: 440 nm), and red emission (λ_{Ex}: 480 nm) of PVK: OLM-TADF-CQDs at solution state with *o*-dichlorobenzene as the solvent.

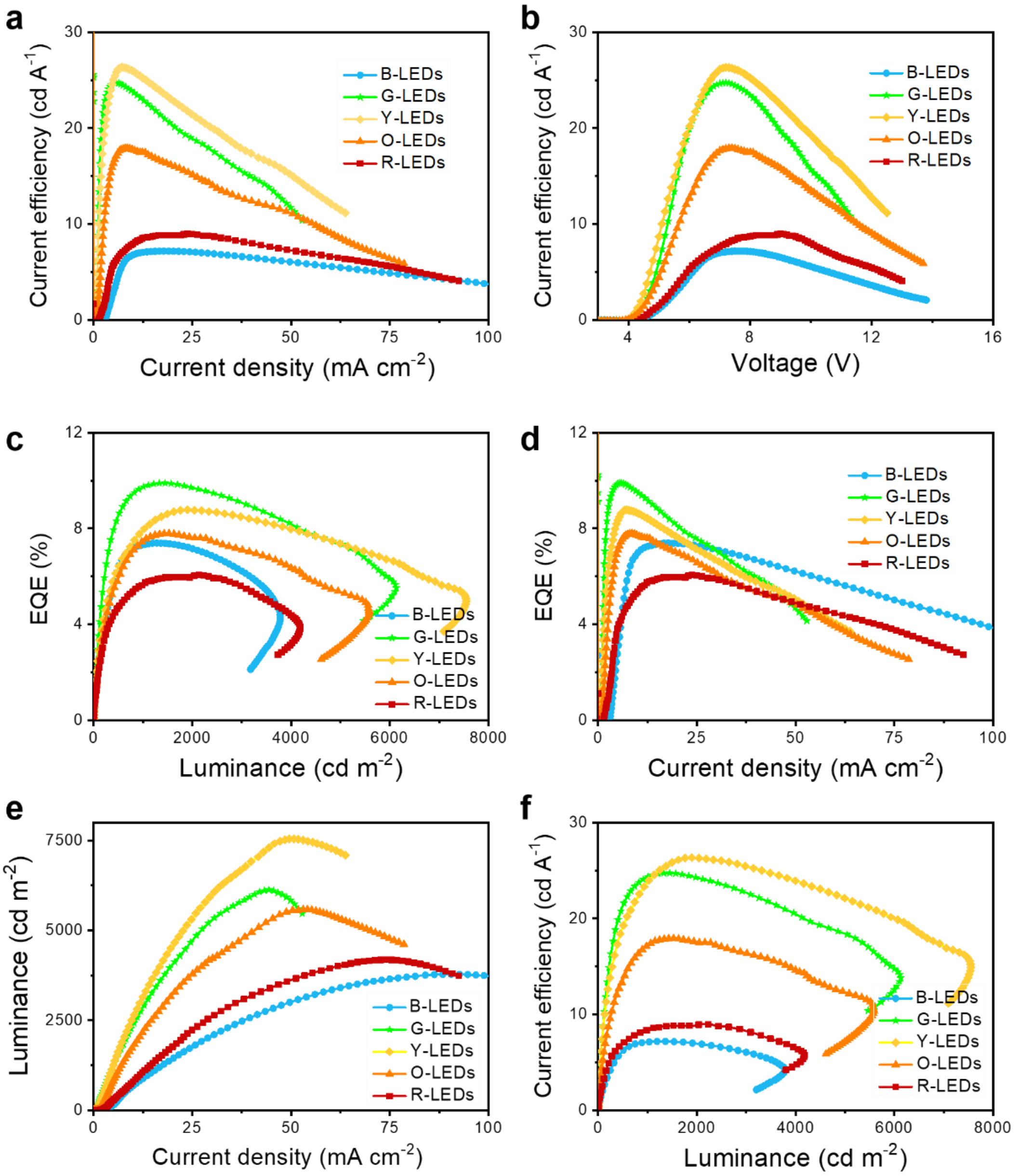

Supplementary Figure 32. Device performance. a Current efficiency-current density, **b** current efficiency-voltage, **c** External quantum efficiency (EQE)-luminance, **d** EQE-current density, **e** luminance-current density, and **f** current efficiency-luminance of light-emitting diodes (LEDs) based on blue (B-), green (G-), yellow (Y-), orange (O-), and red (R-) OLM-TADF-CQDs.

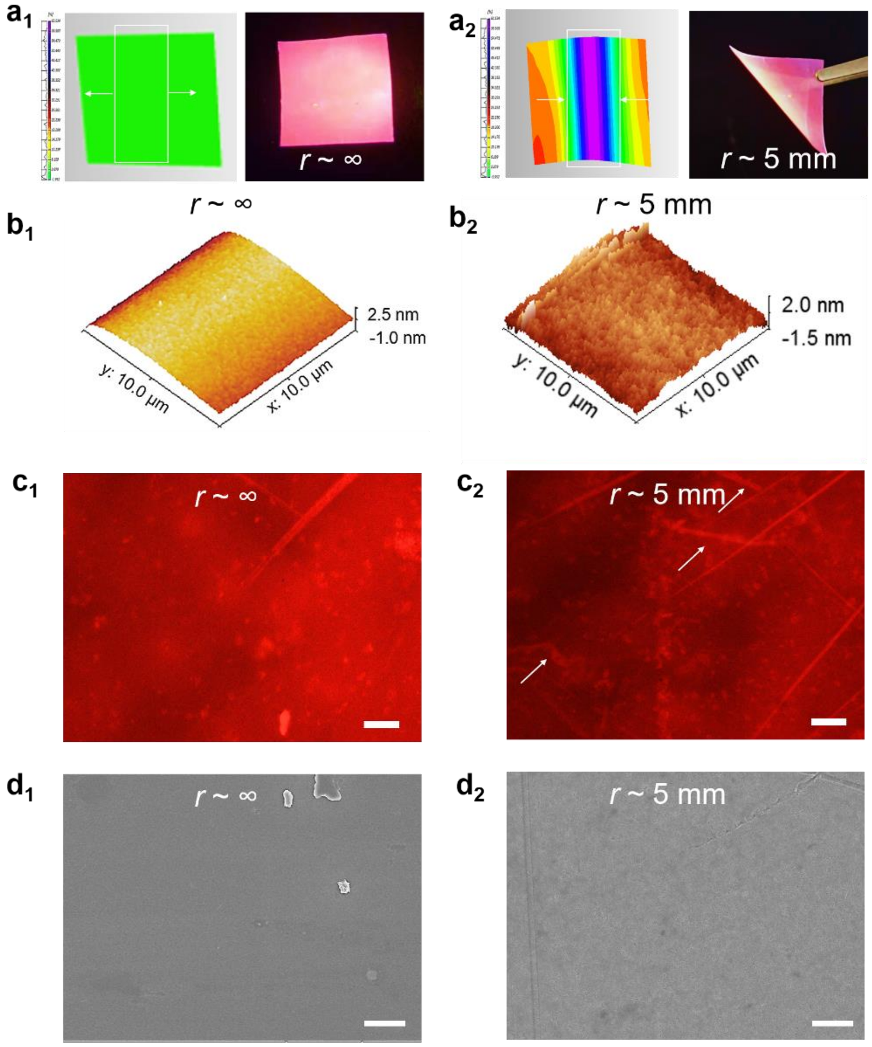

Supplementary Figure 33. The structure and characterizations of flexible film. a_1-a_2 Strain distribution and photographs (at color bare: –1.952–62.534%), **b_1-b_2** AFM images, **c_1-c_2** fluorescence microscope images (Scale bare: 20 μm), and **d_1-d_2** SEM images (Scale bare: 5 μm) of flexible films based on R-OLM-TADF-CQDs under the condition of flat (bending radii (r) ~ ∞) and bent (r ~ 5 mm).

Supplementary Tables

Supplementary Table 1. CV of OLM-TADF-CQDs.

	E_{ox}^{onset} (V)	E_{red}^{onset} (V)	HOMO (eV)	LUMO (eV)
B-OLM-TADF-CQDs	1.00	−1.80	−5.80	−3.00
G-OLM-TADF-CQDs	0.50	−1.90	−5.30	−2.90
Y-OLM-TADF-CQDs	0.30	−1.90	−5.10	−2.80
O-OLM-TADF-CQDs	0.30	−1.80	−5.10	−2.95
R-OLM-TADF-CQDs	0.20	−1.70	−5.00	−3.10

Supplementary Table 2. Photophysical properties of OLM-TADF-CQDs.

	τ_p (ns)	k_p (10^9 s^{-1})	τ_d (μs)	k_d (10^6 s^{-1})	φ_F (%)	φ_{TADF} (%)	φ (%)	k_{ISC} (10^7 s^{-1})	k_{RISC} (10^5 s^{-1})
B-OLM-TADF-CQDs	8.4	0.119	31.2	0.032	2.56	29.44	32	11.595	3.777
G-OLM-TADF-CQDs	9.0	0.111	28.6	0.035	5.26	39.44	44.7	10.516	2.770
Y-OLM-TADF-CQDs	10.5	0.095	25.1	0.040	6.33	31.67	38	8.899	2.136
O-OLM-TADF-CQDs	11.2	0.089	21.9	0.046	11.68	33.32	45	7.860	1.486
R-OLM-TADF-CQDs	11.8	0.085	18.0	0.056	13.14	20.36	33.5	7.383	0.999

Supplementary Table 3. Atomic ratio of monomer CQDs from XPS spectra.

	O (%)	C (%)
B-CQDs	32.04	67.96
G-CQDs	30.78	69.22
Y-CQDs	29.95	70.05
O-CQDs	29.10	70.91
R-CQDs	28.14	71.86

Supplementary Table 4. FWHM of XRD of OLM-TADF-CQDs and several reported carbon dots.

XRD	B-OLM-TADF-CQDs	G-OLM-TADF-CQDs	Y-OLM-TADF-CQDs	O-OLM-TADF-CQDs	R-OLM-TADF-CQDs
FWHM (degree)	12.5	12.5	13.0	13.0	12.0

The title of article		FWHM of XRD (degree)	Reference
Onion-like Multicolor Thermally Activated Delayed Fluorescent Carbon Quantum Dots for Efficient Electroluminescent Light-emitting Diodes	Carbon quantum dots	12.5/12.5/13.0/ 13.0/12.0	This work
Engineering triangular carbon quantum dots with unprecedented narrow bandwidth emission for multicolored LEDs		~8.5	Ref.[1]
Gram-Scale Synthesis of Highly Efficient Rare-Earth Element-Free Red/Green/Blue Solid-State Bandgap Fluorescent Carbon Quantum Rings for White Light-Emitting Diode		~15.5	Ref.[2]
Gram-Scale Synthesis of 41% Efficient Single-Component White-Light-Emissive Carbonized Polymer Dots with Hybrid Fluorescence/Phosphorescence for White Light-Emitting Diodes	Carbonized polymer dots	~13.0	Ref.[3]
Full-color fluorescent carbon quantum dots	Carbon dots	~10.0	Ref.[4]
Deciphering the catalytic mechanism of superoxide dismutase activity of carbon dot nanozyme		~14.5	Ref.[5]
Hydrophobic carbon dots with blue dispersed emission and red aggregation-induced emission		~13.0	Ref.[6]

Supplementary Table 5. Atomic ratio of OLM-TADF-CQDs from XPS spectra.

	O (%)	C (%)
B-OLM-TADF-CQDs	19.85	80.15
G-OLM-TADF-CQDs	16.35	83.65
Y-OLM-TADF-CQDs	14.29	85.71
O-OLM-TADF-CQDs	12.14	87.86
R-OLM-TADF-CQDs	10.60	89.40

Supplementary Table 6. Calculated energy gap and spin-orbit coupling of structure models of monomer CQDs.

Structure models	$\Delta E(S_0/S_1)$ (eV)	$\Delta E(S_0/T_1)$ (eV)	$\Delta E(S_1/T_1)$ (eV)	$\zeta(S_0/S_1)$ (cm^{-1})	$\zeta(S_1/T_1)$ (cm^{-1})
CQDs **1**	2.775	2.401	0.374	0.815	0.112
CQDs **2**	2.288	1.902	0.386	1.808	1.521
CQDs **3**	2.201	1.687	0.514	2.810	0.005
CQDs **4**	2.398	1.605	0.793	1.512	0.007
CQDs **5**	2.218	1.519	0.699	1.909	0.004
CQDs **6**	2.115	1.190	0.925	3.111	0.015
CQDs **7**	1.802	0.904	0.898	2.824	0.155
CQDs **8**	1.821	0.976	0.845	2.709	0.103
CQDs **9**	1.709	0.618	1.091	3.822	0.225

Supplementary Table 7. Calculated energy gap and orbital overlap of structure models of OLM-TADF-CQDs.

Structure models	$\Delta E(S_0/S_1)$ (eV)	$\Delta E(S_1/T_1)$ (eV)	$S_{HOMO\text{-}LUMO}$	
			Ground state	Excited state
OLM-TADF-CQDs **1**	−1.20	−0.68	0.0097	0.0024
OLM-TADF-CQDs **2**	−1.34	−0.89	0.0328	0.0129
OLM-TADF-CQDs **3**	−1.25	−0.82	0.0198	0.0432

Supplementary Methods

Characterization data of monomer CQDs: ^{1}H NMR (400 MHz, DMSO-d_6): δ 8.5–9.5 (homobenzene H) ppm, 6.5–8 (sp^2 domain H) ppm, 5.5–6.5 (perylene-like H) ppm; IR: 1630 (C=O) cm^{-1}, 1240 (C–O) cm^{-1}, 1100 (C–O) cm^{-1}; XPS analysis: C (C=C 284 eV, C–O 285 eV, C=O 288 eV), O (C=O 532 eV, C–O 533 eV).

Characterization data of OLM-TADF-CQDs: ^{1}H NMR (400 MHz, DMSO-d_6): δ 9–11 (perylene-like H), 6–9 (carbonized H) ppm; ^{13}C NMR (400 MHz, DMSO-$d6$): δ 162–178 (perylene-like C) ppm, 120–145 (sp^2 domain C) ppm; IR: 3000-3600 (O–H, C–H) cm^{-1}, 1630 (C=O) cm^{-1}, 1240 (C–O) cm^{-1}; UV-vis: λ_{max} 405 (blue) nm, 456 (green) nm, 484 (yellow) nm, 533 (orange) nm, and 576 (red) nm; PL: λ_{max} 445 (blue) nm, 493 (green) nm, 518 (yellow) nm, 558 (orange) nm, and 605 (red) nm; XPS analysis: C (C=C 284 eV, C=O 289 eV), O (C=O 532 eV, C–O 533 eV).

Quantum Yield Measurements: The QY of OLM-TADF-CQDs is measured using a Varian FLR025 spectrometer combined with a 120 mm integrating sphere. We place the samples in ultraviolet (UV) quartz plates/quartz cuvettes and direct the test-light out of the spectrometer through samples, reaching the sphere with a 10 mm light path. The photons emit and absorb by OLM-TADF-CQDs, and then the QY is obtained A blank quartz plate/quartz cuvette is measured as the reference blank. The spectral correction curve is related to the sensitivity of Edinburgh Instruments.

Ultraviolet Photoelectron Spectroscopy Measurement: The UV photoelectron spectrum of OLM-TADF-CQD thin film is measured with an hv = 21.22 eV, a He I source (AXIS ULTRA DLD, Kratos), an analysis room vacuum of 3.0×10^{-8} Torr, and a bias voltage of −9 V. We prepare the OLM-TADF-CQD films on ITO substrates by spin-coating OLM-TADF-CQDs (5 mg) dissolved in *o*-dichlorobenzene (1 mL).

Characterization Method: A Hitachi SU 8010 scanning electron microscope and a Talos F200S transmission electron microscope (TEM) examine the morphologies of the OLM-TADF-CQDs. A Bruker Dimension FastScan atomic force microscope (AFM) display the morphologies of the film in

tapping mode (Ted Pella-50). Arrow NSC15/AL BS probes (Beijing Xingde Instrument Equipment Co., Ltd.) is used for scanning. X-ray diffraction (XRD) patterns are obtained *via* X-ray diffractometry with Cu-Kα radiation (PANalytical X'Pert Pro MPD). X-ray photoelectron spectroscopy (XPS) is performed using an electron spectrometer with 300 W Al Kα radiation (ESCALab 220i-XL, VG Scientific). Raman spectra are obtained *via* laser confocal micro-Raman spectroscopy (LabRAM Aramis). Fourier transform infrared (FT-IR) spectra are obtained *via* a Thermo Scientific Nicolet 380 spectrometer. A Shimadzu UV-2450 spectrophotometer measures absorption spectra. A PerkinElmer-LS55 luminescence spectrometer with a 2 nm slit width records the fluorescence spectra and the excitation phosphorescence mappings. Time-resolved fluorescence and phosphorescence decay spectra are measured *via* an FLS980 fluorescence spectrofluorometer (Edinburgh). Low-temperature-dependent photoluminescence (PL) spectra are measured from 77 K to 300 K using a liquid nitrogen cooler. A Nikon camera (D7200) captures the photographs of samples under daylight/UV (ENF-280C/FBE, 8 W, 365 nm).

STEM images characterization: A FEI-Themis Z TEM is used to investigate the scanning transmission electron microscopy (STEM) images of the OLM-TADF-CQDs. Ultrathin carbon film supported by a lacey on a 400 mesh copper grid (product no. 01824, Beijing Xinxing Braim Technology Co., Ltd) is used to disperse the CQDs. The purified diluted CQDs ethanol solution with 5 μL is dropped on the surface of ultrathin carbon film and then dried at room temperature. Finally, the STEM images of CQDs samples are taken at 200 kV.

Theoretical calculations: All the energy levels and electron cloud distributions of different model CQDs/OLM-TADF-CQDs are calculated. For the structural models of CQDs, we select a time-dependent density functional theory (TDDFT) method, and combine the 6-311G* basis set and the functional B3LYP (B3LYP/6–311G*). For OLM-TADF-CQDs models, the B3LYP–GD3BJ hybrid functional is employed to predict different stacking structure of two monomer CQDs with weak interaction. Further optimizations of different conformations are carried out for triplet and singlet states at the PBE0/def–TZVP level under vacuum. For all the molecular orbital wavefunctions in the

calculated models, the positive phase is shown as the green in HOMO and LUMO, and the negative phases correspond to blue. The spin-orbit coupling is calculated using the spin-orbit mean-field (SOMF) method.[7,8]

Device Fabrication and Characterization: The indium tin oxide (ITO) glass substrates are prepared through cleaning ultrasonically three times in isopropyl, ethanol, and deionized water. After drying in an oven at 150 °C for 15 min, the cleaned substrates are treated with UV–ozone for 15 min to reduce the surface resistance and obtain ITO with a high work function. The poly(3,4-ethylenedioxythiophene):poly(styrenesulfonate) (PEDOT:PSS) is spin-coated at 2000 rpm for 35 s on the ITO substrate to prepare a hole injection layer. After annealed in an oven at 150 °C for 15 min, the thickness is approximately 30 nm. Next, on the surface of the TFB film, the OLM-TADF-CQDs (5 mg) dissolved in *o*-dichlorobenzene (1 mL) through blending with poly(*N*-vinyl carbazole) (PVK) is spin coated at 3000 rpm for 45 s and dried on a hot plate at 80 °C for 30 min, thus forming the active layer of the OLM-TADF-CQD. The doping ratio of CQDs in the poly(*N*-vinyl carbazole) (PVK) is 5%. PVK is selected as host material due to its excellent hole-transporting properties and favorable film-forming properties After that, by putted into the vacuum evaporation chamber, the substrates are thermally deposited on the 1,3,5-tris(*N*-phenylbenzimidazol-2-yl) benzene (TPBi) at a pressure of 3 × 10^{-4} Pa with a thickness of 30 nm (1 Å s^{-1}). Subsequently, a double-layer cathode is deposited, using Ca and Al with thicknesses of 10 nm (1 Å s^{-1}) and 100 nm (3 Å s^{-1}), respectively. The fabricated devices have an active area of 4 mm^2 at the overlapping area of TPBi and Ca/Al. The HIL PEDOT:PSS as a buffer layer is mainly used to adjust the energy level of the anode ITO from 4.7 eV to 5.0 eV and to stabilize the transmission and injection of holes in the device by reducing the surface roughness and pinhole of the anode-active layer. The electron transport layer (ETL), TPBi, is used to transfer electrons and regulate the interaction of phase interfaces between TPBi and cathode-active emission layer. Energy level matching between layers in devices is the prerequisite for the selection of materials. A Dektak XT (Bruker) surface profilometer and a spectroscopic ellipsometer (Suntech) measure thicknesses of the films. A computer-controlled Keithley 236 SMU and Keithley 200

multimeter equipped with a calibrated Si photodiode record the current density–voltage–luminance (J–V–L) characteristics. Electroluminescence (EL) spectra are obtained using an Ocean Optics 2000 spectrometer, which is coupled with a linear charge-coupled device (CCD) array detector (350–1100 nm).

The following formulas for rate constants are used in this study.[9]

$$k_{\mathrm{p}} = \frac{1}{\tau_{\mathrm{p}}} \qquad (1)$$

$$k_{\mathrm{d}} = \frac{1}{\tau_{\mathrm{d}}} \qquad (2)$$

$$k_{\mathrm{ISC}} = (1 - \emptyset_{\mathrm{F}})k_{\mathrm{p}} \qquad (3)$$

$$k_{\mathrm{RISC}} = \frac{k_{\mathrm{p}}k_{\mathrm{d}}}{k_{\mathrm{ISC}}}\frac{\emptyset_{\mathrm{TADF}}}{\emptyset_{\mathrm{F}}} \qquad (4)$$

$$\emptyset_{\mathrm{ISC}} = 1 - \emptyset_{\mathrm{F}} \qquad (5)$$

$$\emptyset_{\mathrm{RISC}} = \frac{\emptyset_{\mathrm{TADF}}}{\emptyset_{\mathrm{ISC}}} \qquad (6)$$

Where, k_{p} is the prompt rate constant, τ_{p} is the prompt lifetime, k_d is the delay rate constant, τ_{d} is the delay lifetime, k_{ISC} is the intersystem crossing (ISC) rate constant, $\emptyset_{\mathrm{F}}$ is the prompt efficiency, k_{RISC} is the reverse ISC (RISC) rate constant, $\emptyset_{\mathrm{TADF}}$ is the thermally activated delayed fluorescence (TADF) efficiency, k_{F} is the fluorescence rate constant, $\emptyset_{\mathrm{ISC}}$ is the ISC efficiency, $\emptyset_{\mathrm{RISC}}$ is the RISC efficiency.

To extract the important physical parameters of the exciton binding energy, the integrated PL emission intensity as a function of temperature (77–300 K) are plotted. The curves are fitted using the following equation:

$$I(\mathrm{T}) = \frac{I_0}{1+\mathrm{A}e^{-E_{\mathrm{b}}/k_{\mathrm{B}}\mathrm{T}}} \qquad (7)$$

where I_0 denotes the intensity at 0 K, E_{b} indicates the exciton binding energy, and k_{B} represents the Boltzmann constant.[10]

Supplementary References

1. Yan, F. L., et al. Engineering triangular carbon quantum dots with unprecedented narrow bandwidth emission for multicolored LEDs. *Nat. Commun.* **9**, 2249 (2018).
2. Meng, T., et al. Gram-scale synthesis of highly efficient rare-earth element-free red/green/blue solid-state bandgap fluorescent carbon quantum rings for white light-emitting diode. *Angew. Chem. Int. Ed.* **60**, 16343–16348 (2021).
3. Wang, Z. F., et al. Gram-scale synthesis of 41% efficient single-component white-light-emissive carbonized polymer dots with hybrid fluorescence/phosphorescence for white light-emitting diodes. *Adv. Sci.* **7**, 1902688 (2020).
4. Wang, L., et al. Full-color fluorescent carbon quantum dots. *Sci. Adv.* **6**, eabb6772 (2020).
5. Gao, W. H., et al. Deciphering the catalytic mechanism of superoxide dismutase activity of carbon dot nanozyme. *Nat. Commun.* **14**, 160 (2023).
6. Yang, H. Y., et al. Hydrophobic carbon dots with blue dispersed emission and red aggregation-induced emission. *Nat. Commun.* **10**, 1789 (2019).
7. Witte, J., Goldey, M., Neaton, J. B. & Head-Gordon, M. Beyond energies: Geometries of nonbonded molecular complexes as metrics for assessing electronic structure approaches. *J. Chem. Theory Comput.* **11**, 1481–1492 (2015).
8. Frisch, M. J., et al. Gaussian 09, rev. B. 01; Gaussian. Inc.: Wallingford, CT, (2009).
9. Lee, H., Chung, W. & Lee, J. Efficient up-conversion process by isolation of two chromophores in thermally activated delayed fluorescent emitters. *Chem. Eng. J.* **409**, 128285 (2021).
10. Yuan, F. L., et al. Bright high-colour-purity deep-blue carbon dot light-emitting diodes via efficient edge amination. *Nat. Photonics* **14**, 171–187 (2020).
