# Supplementary material for: Onion-like multicolor thermally activated delayed fluorescent carbon quantum dots for efficient electroluminescent light-emitting diodes
Source: Nat Commun. 2024 Apr 8;15:3043. doi: 10.1038/s41467-024-47372-8 (PMC11001924; doi:10.1038/s41467-024-47372-8)
Supplement: Supplementary file 3 — Description of Additional Supplementary Files [file 41467_2024_47372_MOESM3_ESM.pdf]

Description of Additional Supplementary Files

File name: Supplementary Data 1

Description: Optimized cartesian coordinates of CQDs 1. The cartesian coordinates (Å) include of CQDs **1** for the ground state, the first excited singlet state, and the first excited triplet state.

File name: Supplementary Data 2

Description: Optimized cartesian coordinates of OLM-TADF-CQDs 1. Optimized Cartesian coordinates (Å) of OLM-TADF-CQDs **1** for the ground state and for the excited state.

File name: Supplementary Movie 1

Description: 3D construction of OLM-TADF-CQDs. STEM image reconstruction of serial tilts (α from –70° to +70°) using the simultaneous iterative reconstruction technique, in which the 3D structure and different layers of OLM-TADF-CQDs are visualized with false color.
